# The E3 ubiquitin ligase MARCH2 protects against myocardial ischemia-reperfusion injury through inhibiting pyroptosis via negative regulation of PGAM5/MAVS/NLRP3 axis

**DOI:** 10.1038/s41421-023-00622-3

**Published:** 2024-02-27

**Authors:** Shuolin Liu, Yaguang Bi, Tianting Han, Yiran E. Li, Qihang Wang, Ne Natalie Wu, Chenguo Xu, Junbo Ge, Ronggui Hu, Yingmei Zhang

**Affiliations:** 1 Department of Cardiology, Zhongshan Hospital, Fudan University, Shanghai Institute of Cardiovascular Diseases, National Clinical Research Center for Interventional Medicine, Key Laboratory of Viral Heart Diseases, National Health Commission. Key Laboratory of Viral Heart Diseases, Chinese Academy of Medical Sciences, Shanghai, China; 2 College of Basic Medicine, Shanghai Medical College, Fudan University, Shanghai, China; 3 State Key Laboratory of Molecular Biology, Center for Excellence in Molecular Cell Science, Shanghai Institute of Biochemistry and Cell Biology, Chinese Academy of Sciences, Shanghai, China; 4 University of Chinese Academy of Sciences, Beijing, China; 5 School of Life Science, Hangzhou Institute for Advance Study, University of Chinese Academy of Sciences, Hangzhou, Zhejiang China

**Keywords:** Ubiquitylation, Mechanisms of disease, Necroptosis

## Abstract

Inflammasome activation and pyroptotic cell death are known to contribute to the pathogenesis of cardiovascular diseases, such as myocardial ischemia-reperfusion (I/R) injury, although the underlying regulatory mechanisms remain poorly understood. Here we report that expression levels of the E3 ubiquitin ligase membrane-associated RING finger protein 2 (MARCH2) were elevated in ischemic human hearts or mouse hearts upon I/R injury. Genetic ablation of *MARCH2* aggravated myocardial infarction and cardiac dysfunction upon myocardial I/R injury. Single-cell RNA-seq analysis suggested that loss of MARCH2 prompted activation of NLRP3 inflammasome in cardiomyocytes. Mechanistically, phosphoglycerate mutase 5 (PGAM5) was found to act as a novel regulator of MAVS-NLRP3 signaling by forming liquid-liquid phase separation condensates with MAVS and fostering the recruitment of NLRP3. MARCH2 directly interacts with PGAM5 to promote its K48-linked polyubiquitination and proteasomal degradation, resulting in reduced PGAM5–MAVS co-condensation, and consequently inhibition of NLRP3 inflammasome activation and cardiomyocyte pyroptosis. AAV-based re-introduction of MARCH2 significantly ameliorated I/R-induced mouse heart dysfunction. Altogether, our findings reveal a novel mechanism where MARCH2-mediated ubiquitination negatively regulates the PGAM5/MAVS/NLRP3 axis to protect against cardiomyocyte pyroptosis and myocardial I/R injury.

## Introduction

The inflammasome is a macromolecular structure responsible for sensing injury and eliciting a cascade of inflammatory responses. The most well-characterized sensor of inflammasomes is NACHT, LRR, and PYD domains-containing protein 3 (NLRP3), which is activated in response to sterile inflammation^1^. Activation of inflammasome sensor NLRP3 recruits the adaptor apoptosis-associated speck-like protein containing a caspase-recruitment domain (ASC) and the effector caspase-1, triggering severe tissue damage directly by promoting pyroptosis and indirectly by IL-18 secretion^2,3^. NLRP3 inflammasome activation contributes to the pathophysiology of numerous inflammatory diseases^4^. For example, NLRP3 inflammasome activated by damage-associated molecular patterns (DAMPs) participates in the pathogenesis of nonalcoholic fatty liver disease^5^. Additionally, expression levels of NLRP3 and inflammatory cytokines IL-18 and IL-1β were found to be elevated in atherosclerosis plaque development^6^. Besides, knockout (KO) of NLRP3 protects against high-fat diet-elicited obesity and insulin resistance by abrogating reactive oxygen species (ROS)- and mitochondrial injury-evoked inflammasome activation^7^.

Myocardial infarction (MI) remains one of the leading causes of disability and mortality worldwide^8^. Timely restoration of blood flow through occluded coronary arteries represents an optimal maneuver for patients with myocardial infarction^9^. However, such reperfusion may activate cascades of detrimental injuries to the myocardium known as ischemia-reperfusion (I/R) injury^9,10^. Emerging evidence has indicated the activation of inflammasomes as an integral part of inflammation-driven myocardial I/R injury^1,11^. Suppressing NLRP3 inflammasome activation is known to restrict infarct size and cardiac dysfunction following myocardial I/R injury^12,13^. Therefore, the identification of endogenous modulator(s) for NLRP3 inflammasome to suppress its activation might be clinically beneficial.

The ubiquitin (Ub)-proteasome system regulates protein degradation and homeostasis in numerous cellular processes^14^. Therefore, identifying pivotal ubiquitination-related modulators may alleviate myocardial I/R injury. For this purpose, we performed single-cell RNA sequencing (scRNA-seq) on heart samples from mice with myocardial I/R injury. We found upregulation of the E3 ligase membrane-associated RING finger protein 2 (MARCH2), a member of the MARCH E3 Ub ligase subfamily including 11 members and localizing primarily to the endoplasmic reticulum, endosome, Golgi apparatus, mitochondria, and plasma membranes^15^. MARCH2 is known to regulate vesicular trafficking between the endosomes and trans-Golgi network as well as recycle endosomes through interacting with syntaxin-6^16^. MARCH2 was also shown to negatively regulate autophagy in tumor cells by promoting ubiquitination and degradation of CFTR and activating the PIK3CA-AKT-mTOR pathway^17^.

Recent evidence has demonstrated an essential role for MARCH2 in the regulation of inflammatory responses, such as antiviral defense and bacterial infection, as well as T-cell development^18^. MARCH2 is upregulated upon HIV infection, which restricts the production and infection of HIV-1^19^. In addition, MARCH2 catalyzes K48-linked ubiquitination on NF-κB essential modulator, a critical modulator functioning in NF-κB and interferon-mediated signaling. *MARCH2* KO leads to robust production of proinflammatory cytokines (IL-6, CXCL10, TNF-α and IL-1β) upon lipopolysaccharides (LPS) challenge^20^. Nevertheless, whether MARCH2 plays a role in myocardial I/R injury remains elusive.

Taking advantage of the *MARCH2* KO and AAV9-cTnT-MARCH2 transfection murine models, our present study showed that MARCH2 ameliorates myocardial I/R injury and suppresses NLRP3 inflammasome activation. MARCH2 was found to interact with phosphoglycerate mutase family member 5 (PGAM5) to promote its K48-linked ubiquitination and degradation. Ubiquitin-dependent degradation of PGAM5 leads to inhibition of phase-separated condensates of PGAM5-mitochondrial anti-viral-signaling protein (MAVS) and activation of downstream NLRP3 inflammasome. Taken together, these findings demonstrate the vital role of MARCH2 as a novel therapeutic target for myocardial I/R injury.

## Results

### Characterization of MARCH2 expression during myocardial I/R

**Fig. 1:**
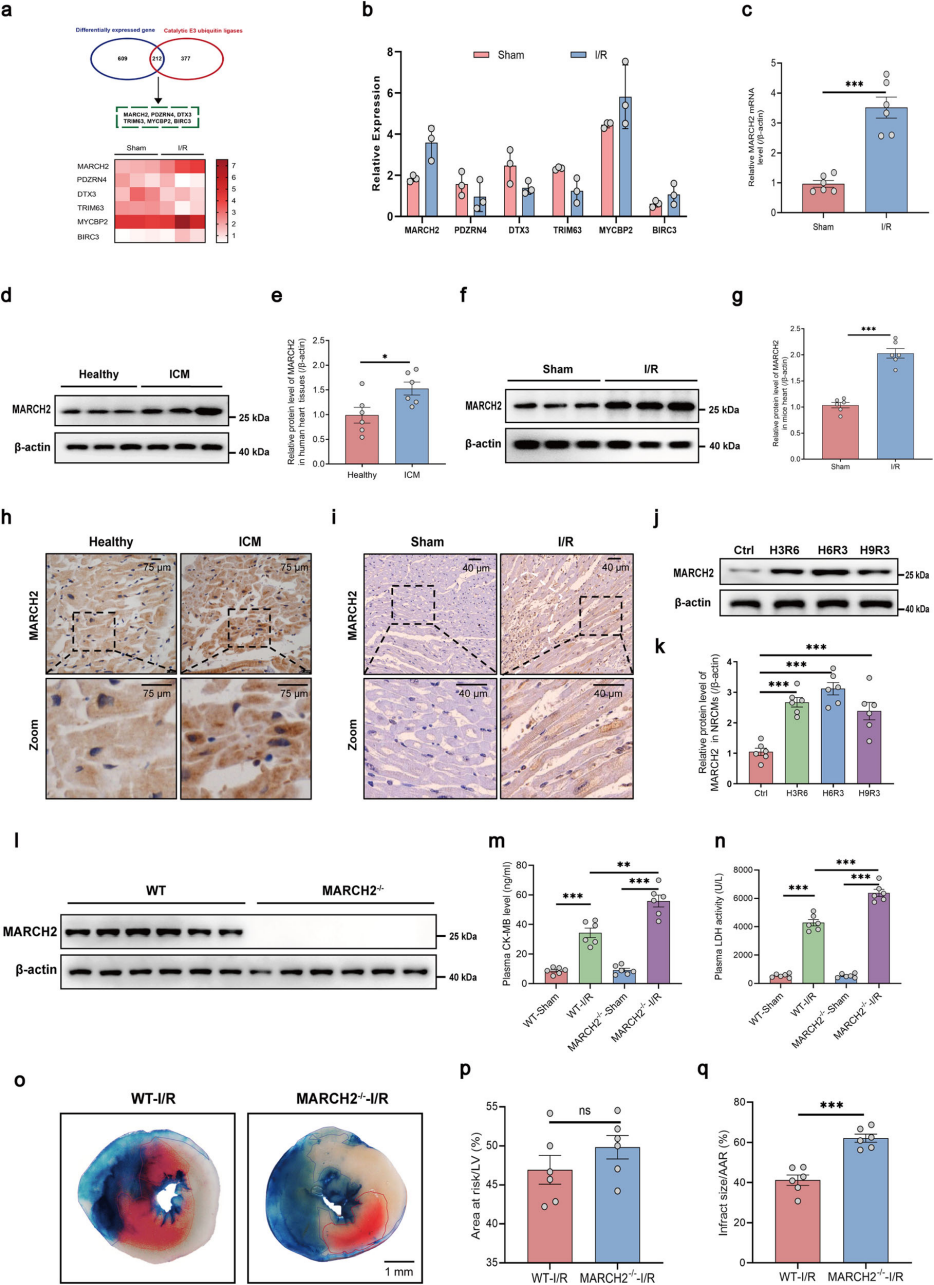
MARCH2 protein level is increased in hearts of patients with ICM and mice during myocardial I/R. **a** Heatmap showing six differentially expressed E3 ligases in mouse hearts following I/R injury. **b** Histogram showing the relative mRNA expression of the six ubiquitin-related genes in the indicated groups. **c** mRNA levels of MARCH2 following I/R injury (45 min/9 h) were analyzed by RT-qPCR (normalized to β-actin). **d** Western blotting analysis of MARCH2 expression in healthy human hearts and patients with ICM. **e** Quantitated MARCH2 levels in hearts of healthy human and patients with ICM (*n* = 6 for each group). **f** Western blotting analysis of MARCH2 protein levels following I/R injury (45 min/9 h). **g** Quantitated MARCH2 levels following I/R injury (45 min/9 h). **h** Representative immunohistochemistry images showing MARCH2 protein levels in human hearts. **i** Representative immunohistochemistry images showing MARCH2 protein levels in sham- or I/R-treated mouse hearts. Dotted white lines indicate the boundary of the infarct area and border area. **j**, **k** Representative western blotting image (**j**) and quantification analysis (**k**) of MARCH2 expression in NMCMs subjected to hypoxia (6 h)/reoxygenation (3 h) (*n* = 6 for each group). **l** Western blotting analysis showed that MARCH2 protein could not be detected following the deletion of *MARCH2* gene. **m**, **n** CK-MB level (**m**) and LDH activity (**n**) of WT and *MARCH2* KO mice with or without myocardial I/R (45 min/24 h) (*n* = 6 for each group). **o** Representative images of heart sections by TTC/Evans Blue staining depicting infracted area. **p** Ratios of area at risk (AAR) to left ventricular (LV) area. **q** Infarct area normalized to AAR. Data are shown as means ± SEM. Statistical significance was examined by two-way ANOVA with Bonferroni post-test. *P < 0.05, **P < 0.01, ***P < 0.001.

To reveal possible ubiquitination-related candidate molecules that might participate in the pathogenesis of myocardial I/R injury, we performed scRNA-seq for wild-type (WT) mice subjected to sham or I/R surgery. Upregulated or downregulated differentially expressed genes (DEGs) were screened (Supplementary Fig. S1a). We found that six E3 ligases reached the threshold of screening, namely *MARCH2*, *PDZRN4*, *DTX3*, *TRIM63*, *MYCBP2*, and *BIRC3*, among which *MARCH2* exhibited the most pronounced transcriptional change (Fig. 1a, b). To confirm the results of scRNA-seq, we next performed qPCR analyses and found that mRNA levels of *MARCH2* was increased following I/R injury (Fig. 1c), suggesting a potential role of *MARCH2* in myocardial I/R injury. Levels of MARCH2 were evaluated in heart tissues from patients with ischemic cardiomyopathy (ICM) (Supplementary Table S1). Western blotting and immunohistochemistry data revealed elevated MARCH2 levels in heart tissues from ICM patients in comparison with healthy controls (Fig. 1d, e, h). Consistently, western blotting and immunohistochemistry results revealed increased myocardial MARCH2 levels following I/R injury (45 min/9 h) in mice (Fig. 1f, g, i). A similar pattern was observed in primary neonatal mouse cardiomyocytes (NMCMs) and primary adult mouse cardiomyocytes during hypoxia and reoxygenation (H/R) (Fig. 1j, k; Supplementary Fig. S1b). Altogether, these data indicate an integral role for MARCH2 in myocardial I/R injury.

### *MARCH2* deficiency exacerbates cardiac dysfunction during I/R injury

To elucidate potential role of MARCH2 in I/R injury, *MARCH2* KO mice were generated and genotype was confirmed (Fig. 1l; Supplementary Fig. S2a). *MARCH2* homozygous KO mice showed normal baseline heart rate and systolic blood pressure comparable to those from heterozygous or WT mice (Supplementary Fig. S2b, c). WT and *MARCH2* KO mice were then subjected to I/R surgery. Myocardial I/R challenge increased levels of creatine phosphokinase-MB (CK-MB) and lactic dehydrogenase (LDH), two cardiac injury markers, with more pronounced rises in *MARCH2* KO mice (Fig. 1m, n). Evans blue/TTC double staining revealed comparable area at risk (AAR) between WT and *MARCH2* KO mice following myocardial I/R (45 min/24 h), with a more pronounced infarct size response in *MARCH2* KO mice (Fig. 1o–q).

**Fig. 2:**
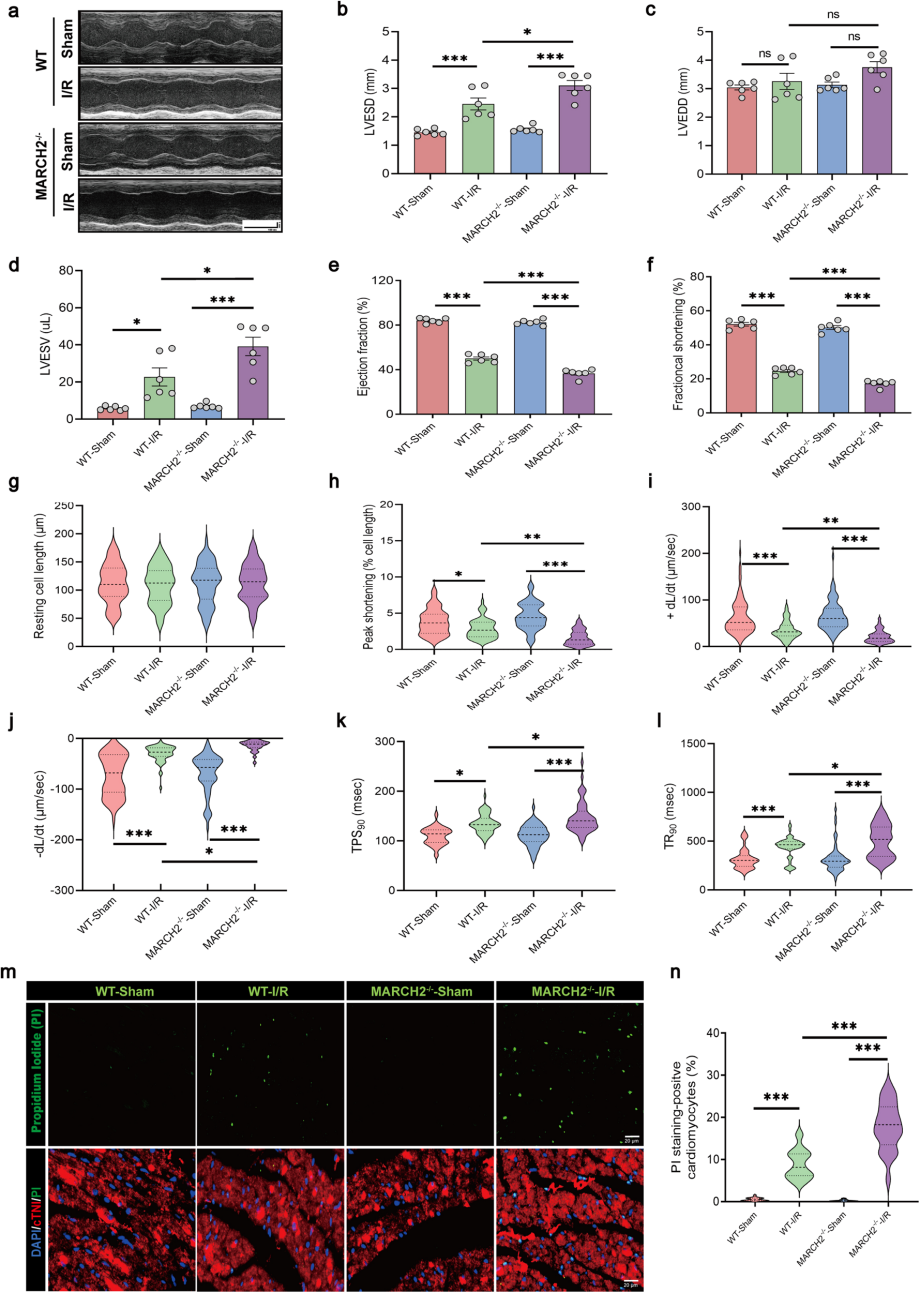
*MARCH2* KO exacerbates myocardial dysfunction after myocardial I/R injury. **a** Representative M-mode recordings of echocardiography of WT and *MARCH2* KO mice subjected to I/R. **b**–**f** Quantitative analyses of echocardiographic measurements performed after I/R injury. *n* = 6 mice per group. Left ventricular end-systolic diameter (LVESD, **b**), left ventricular end-diastolic diameter (LVEDD, **c**), left ventricular end-systolic volume (LVESV, **d**), ejection fraction (EF, **e**), and fractional shortening (FS, **f**). **g**–**l** Evaluation of mechanical properties of single, acutely isolated cardiomyocytes from I/R-treated mice. Resting cell length (**g**), peak shortening (**h**), +dL/dt: maximal velocity of shortening (**i**), -dL/dt: maximal velocity of re-lengthening (**j**), TPS_90_: time-to-90% peak shortening (**k**); TR_90_: time-to-90% re-lengthening (**l**). *n* = 60 cells from 3 mice per group. **m** Representative PI-stained images of myocardial sections from WT and *MARCH2*^–/–^ mice subjected to I/R. Green: PI-positive nuclei; Red: cTnI-stained cardiomyocytes; blue, DAPI-stained nuclei; Scale bar = 20 μm. **n** Quantitative analysis of PI-positive cells. Experiments were repeated three times with similar results. Data are shown as the means ± SEM. *P < 0.05, **P < 0.01, ***P < 0.001.

Echocardiographic analysis showed comparable cardiac function and morphology between *MARCH2* KO and WT mice under physiological conditions. I/R procedure evoked markedly decreases in left ventricular ejection fraction (LVEF), left ventricular fractional shortening (LVFS), and increases in left ventricular end-systolic diameter (LVESD), left ventricular end-systolic volume (LVESV) in WT mice, and these dysfunctions were accentuated by *MARCH2* KO (Fig. 2a–f). Chronological echocardiographic assessment of cardiac structure and function was performed. The aggravation caused by *MARCH2* KO sustained from 24 h after reperfusion to 28 days (Supplementary Fig. S2d–g).

Contractile properties of adult cardiomyocytes isolated from sham or I/R-treated mice were evaluated. Neither I/R procedure nor *MARCH2* ablation exerted any effect on resting cardiomyocyte length (Fig. 2g). However, I/R injury-evoked suppression in peak shortening and maximal velocity of shortening/re-lengthening (±dL/dt) as well as prolonged shortening duration (TPS_90_) and prolonged re-lengthening duration (TR_90_). *MARCH2* ablation markedly aggravated I/R-induced cardiomyocyte contractile anomalies without eliciting any notable effect itself (Fig. 2h–l). Besides, cardiomyocyte necrosis was monitored using propidium iodid (PI) staining. The PI-positive cardiomyocytes were notably increased following I/R procedure, which was significantly exacerbated in *MARCH2* KO mice (Fig. 2m, n). These data suggest that *MARCH2* deficiency accentuates I/R-elicited myocardial dysfunction and cardiomyocyte death.

### *MARCH2* deletion induces NLRP3 inflammasome activation following myocardial I/R

**Fig. 3:**
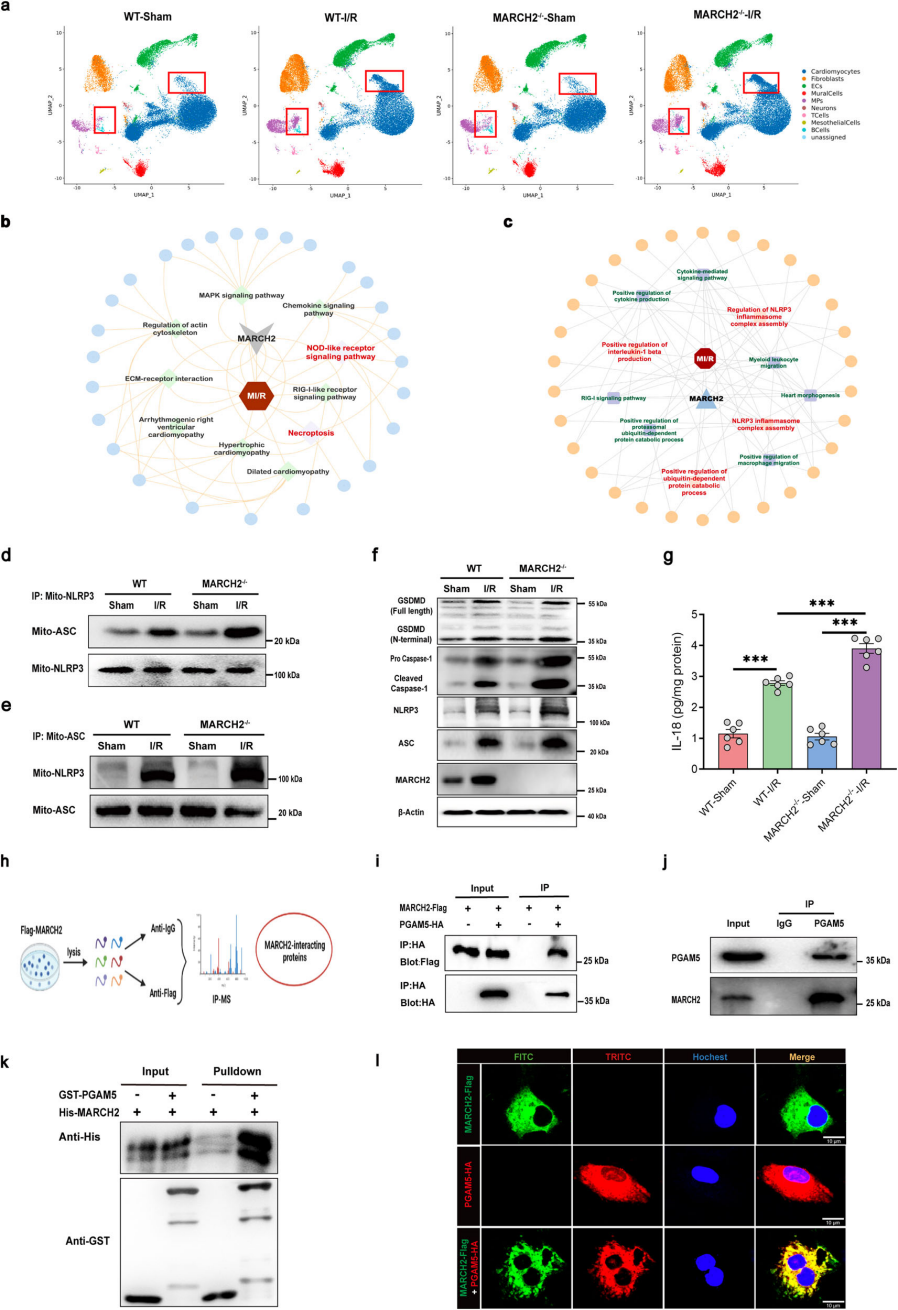
PGAM5 is identified to interact with MARCH2. **a** Comparison of cell type distributions among different groups showing changes in MP and cardiomyocyte clusters after I/R procedure. **b**, **c** KEGG enrichment analysis (**b**) and GO (**c**) analysis of scRNA-seq data from WT-I/R and *MARCH2*^–/–^-I/R hearts. **d**, **e** Interaction between NLRP3 and ASC in mitochondria (mito-NLRP3 and mito-ASC) of cardiac tissues of *MARCH2* KO and WT mice subjected to I/R was examined by IP-western blotting assay. IP with NLRP3 antibody (**d**) and IP with ASC antibody (**e**). **f** Representative Western blotting analyses of MARCH2, NLRP3, ASC, caspase-1 (procaspase1; cleaved caspase-1), GSDMD (full-length; N-terminal) in cardiac tissues of *MARCH2* KO and WT mice subjected to I/R (45 min/9 h). **g** IL-18 release was measured by enzyme-linked immunosorbent assay (ELISA) in cardiac tissues of *MARCH2* KO and WT mice subjected to I/R (45 min/9 h). **h** Schematic diagram showing MS analysis workflow for identifying targets of MARCH2. **i** IP analysis with anti-HA antibody and immunoblotting with antibodies of anti-Flag and anti-HA, respectively, in NMCMs transfected with PGAM5-HA or control vector along with MARCH2-Flag. **j** Endogenous IP of MARCH2 and PGAM5 in NMCMs. **k** Direct interaction between GST-PGAM5 and His-MARCH2 demonstrated by GST pull-down assays. Both input and pull-down samples were immunoblotted with anti-GST and anti-His antibodies. GST-PGAM5 and His-MARCH2 proteins were expressed in vitro. **l** Representative immunofluorescence images of MARCH2 and PGAM5 in NMCMs. Data are shown as the means ± SEM. *P < 0.05, **P < 0.01, ***P < 0.001.

To explore the mechanisms through which *MARCH2* KO accentuates cardiac I/R injury, scRNA-seq was performed in WT and *MARCH2* KO mice that underwent sham or I/R surgery. Uniform Manifold Approximation and Projection (UMAP) analysis of scRNA-seq data (*n* = 178,274 cells) revealed cell types including cardiomyocytes, fibroblasts, endothelial cells (ECs), mural cells, macrophages (MPs), neurons, T cells, mesothelial cells, and B cells (Supplementary Fig. S3a–c). Cell type distribution comparison among four groups revealed significant changes in MP and cardiomyocyte clusters after the I/R procedure (Fig. 3a). WT-I/R group exhibited significant upregulation of NLRP3 inflammasome assembly-related genes in B cells, cardiomyocytes, ECs, fibroblasts, and MPs compared with WT-sham group, indicating a role of NLRP3 inflammasome assembly in myocardial I/R injury response (Supplementary Fig. S3d).

Kyoto Encyclopedia of Genes and Genomes (KEGG) analysis found that genes involved in the NOD-like receptor signaling pathway, necroptosis, hypertrophic cardiomyopathy, and dilated cardiomyopathy were upregulated in hearts of *MARCH2* KO mice subjected to I/R injury (Fig. 3b). Gene ontology (GO) analysis revealed NLRP3 inflammasome complex assembly, positive regulation of cytokine production, and positive regulation of ubiquitin-dependent protein catabolic process as the top enriched pathways in *MARCH2* KO-I/R mice compared with WT-I/R group (Fig. 3c), suggesting an essential role of MARCH2 in the regulation of inflammatory response. To further discern the cell types involved in *MARCH2* deficiency-elicited responses, expression levels of essential genes in this signaling cascade including *NLRP3*, *caspase-1*, *IL-18* and *GSDMD* were examined in *MARCH2* KO-I/R and WT-I/R groups. Our data showed that *MARCH2* KO exhibited higher expression levels of these genes primarily in cardiomyocytes (Supplementary Fig. S4). Furthermore, comparative gene set variation analysis (GSVA) also suggested significant upregulation of NOD-like receptor (Supplementary Fig. S5a) and NLRP3 inflammasome complex assembly (Supplementary Fig. S5b) in *MARCH2* KO-I/R cardiomyocytes compared with WT-I/R cardiomyocytes. Next, we verified the effect of *MARCH2* on NLRP3 inflammasome complex assembly signaling. As shown in Fig. 3d, interaction between NLRP3 and the inflammasome adaptor protein ASC in mitochondria was increased in *MARCH2* KO mice compared to WT mice following I/R injury. Consistently, ASC interaction with NLRP3 was enhanced in *MARCH2* KO mice upon I/R stress (Fig. 3e). Expression levels of NLRP3 and ASC were upregulated in the hearts of WT mice subjected to I/R surgery, with a more pronounced response in *MARCH2* KO mice (Fig. 3f; Supplementary Fig. S6a, b). Moreover, increased caspase-1 and GSDMD cleavage (Fig. 3f; Supplementary Fig. S6c, d) and downstream proinflammatory IL-18 secretion after I/R (Fig. 3g) were intensified by *MARCH2* KO.

### MARCH2 directly interacts with PGAM5

In an effort to elucidate the molecular mechanism of MARCH2 in the regulation of myocardial I/R injury, we screened for MARCH2-binding proteins using immunoprecipitation-mass spectrometry (IP-MS) (Fig. 3h; Supplementary Fig. S7a), and focused on genes involved in both necroptosis and NOD-like receptor signaling pathway. Among the precipitated proteins, PGAM5 is implicated in NOD-like receptor signaling pathway and inflammasome activation^21,22^. Deletion of *PGAM5* attenuated I/R-induced necroptosis-like cell death and inflammation response^23^. To investigate whether MARCH2 protects against I/R injury and negatively regulates NLRP3 inflammasome complex assembly via a direct interaction with PGAM5, a co-IP assay was performed. NMCMs were transfected with HA-tagged PGAM5 or control vector along with Flag-tagged MARCH2. Flag-tagged MARCH2 was found in the HA-PGAM5 IP complexes (Fig. 3i). More importantly, co-IP in NMCMs revealed that endogenous MARCH2 interacts with PGAM5 but not control IgG (Fig. 3j). Furthermore, purified glutathione S-transferase (GST)-fused PGAM5 was capable of pulling down MARCH2 (Fig. 3k), suggesting a direct interaction between PGAM5 and MARCH2. Consistently, immunofluorescence showed colocalization between Flag-MARCH2 and HA-PGAM5 in NMCMs (Fig. 3l). These results suggest a direct interaction between MARCH2 and PGAM5 in cardiomyocytes under physiological conditions.

### MARCH2 mediates ubiquitination and proteasomal degradation of PGAM5 by catalyzing K48-linked polyubiquitination

**Fig. 4:**
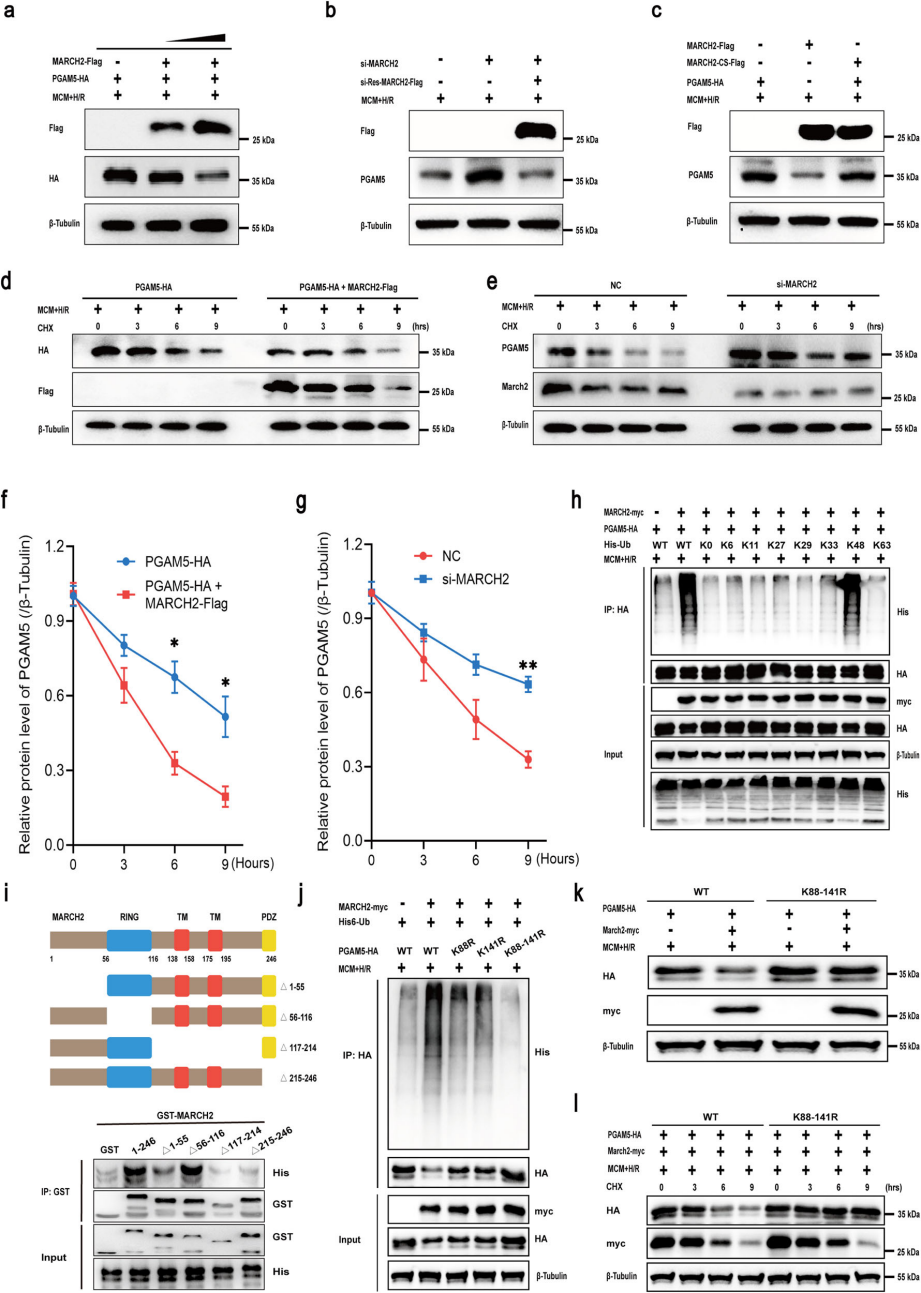
MARCH2 E3 ligase promotes degradation of PGAM5 through K48-linked polyUb. **a** Western blotting analysis of PGAM5-HA and MARCH2-Flag. HL-1 cardiomyocytes were transfected with vector expressing PGAM5-HA or MARCH2-Flag and treated with MCM + H/R. **b** Western blotting analysis of MARCH2 and PGAM5 proteins in HL-1 cardiomyocytes transfected with *MARCH2* siRNA, siRNA-resistant *MARCH2* or negative control and treated with MCM + H/R. **c** Analysis of PGAM5-HA protein levels in MCM + H/R-treated HL-1 cardiomyocytes transfected with either MARCH2 WT or its catalytically inactive mutant (C64/67 S, abbreviated CS). **d**, **e** Western blotting analysis of MARCH2 and PGAM5 protein levels and quantification analysis of PGAM5 level. HL-1 cardiomyocytes were transfected with MARCH2 (**d**) or siRNA-*MARCH2* (**e**) and then treated with MCM + H/R and cycloheximide (CHX; 30 μm). **f**, **g** Corresponding quantitation graphs of relative PGAM5 degradation in MCM + H/R-treated HL-1 cardiomyocytes transfected with MARCH2 (**f**) or siRNA-*MARCH2* (**g**) and CHX. The experiments were repeated three times. Error bars represent standard deviation. **h** Effects of the indicated polyUb on MARCH2-mediated PGAM5 ubiquitination. HL-1 cardiomyocytes were transfected with the indicated ubiquitin under MCM + H/R treatment. IP analysis with anti-HA antibody and immunoblotting with antibodies of anti-His and anti-HA. **i** Western blotting of the mapping analysis showing the binding domains of MARCH2 to PGAM5. Various truncated forms of MARCH2 (△1–55, △56–116, △117–214 and △215–246) were expressed and purified. **j**–**l** Effects of the indicated PGAM5 KR mutants (K88R, K141R, or combined K88-141R) on MARCH2-mediated PGAM5 ubiquitination (**j**), protein level (**k**), and degradation (**l**) under MCM + H/R treatment. PGAM5^–/–^ HL-1 cardiomyocytes were transfected with the indicated constructs, and PGAM5 protein levels were analyzed. Statistical differences were analyzed by unpaired Student’s *t*-test for comparison between two groups. *P < 0.05, **P < 0.01.

Cell–cell interactions in hearts of WT and *MARCH2* KO mice subjected to I/R were predicted using Cellphone DB. As shown in Supplementary Fig. S7b, interaction scores between MPs and cardiomyocytes were increased in *MARCH2* KO-I/R hearts compared with WT-I/R group. To mimic H/R injury and proinflammatory environment in vitro, cardiomyocytes were treated with macrophage-conditioned medium (MCM) and H/R (6 h/3 h). Given the biochemical property of MARCH2 as an E3 Ub ligase, we tested whether PGAM5 may serve as a previously unidentified substrate of MARCH2. HL-1 cardiomyocytes were transfected with plasmids expressing MARCH2-Flag and PGAM5-HA or transfected with empty plasmid vectors (as control) prior to MCM + H/R challenge. Levels of PGAM5 were downregulated in a dose-dependent manner following rises in MARCH2 protein levels in MCM + H/R-treated cardiomyocytes (Fig. 4a), whereas knockdown of MARCH2 upregulated PGAM5 levels and the siRNA-resistant *MARCH2* exhibited an opposite effect (Fig. 4b). Nonetheless, knockdown or overexpression of MARCH2 did not affect the mRNA level of PGAM5 in MCM + H/R-exposed NMCMs (Supplementary Fig. S8a, b). Notably, transfection of a catalytically inactive mutant (C64/67 S, abbreviated as CS) of *MARCH2* abolished MARCH2-accelerated degradation of PGAM5 (Fig. 4c), suggesting an important effect of catalytic activity of MARCH2 in decreasing PGAM5 protein level in the setting of MCM + H/R insult. The degradation dynamics of PGAM5 was examined following cycloheximide (CHX) treatment for various durations. Overexpression of MARCH2 significantly promoted degradation of PGAM5 in comparison to that in control (Fig. 4d, f), while knockdown of MARCH2 exhibited the opposite effect in MCM + H/R-treated HL-1 cardiomyocytes (Fig. 4e–g) and NMCMs under MCM + H/R treatment (Supplementary Fig. S8c). Consistently, the proteasome inhibitor MG132 partially reversed PGAM5 level under MARCH2 overexpression in MCM + H/R-treated NMCMs (Supplementary Fig. S8d). Together, these data suggest that MARCH2 promotes proteasome-dependent degradation of PGAM5.

Furthermore, WT MARCH2 but not the E3 ligase-inactive mutant mediated PGAM5 polyubiquitination (polyUb) upon MCM + H/R treatment (Supplementary Fig. S9a). PolyUb chains of different Lys (K) linkages are known to confer distinct signaling outcomes^24^. To determine the linkage type of the polyUb chains that MARCH2 conjugates onto PGAM5, a series of Ub mutants were generated to preserve Lys on specific residues (Kx = K6, K11, K27, K29, K33, K48, and K63, with the other Lys residues mutated into Arginine (Arg), while K0 refers to the Ub mutant with all Lys substituted with Arg thus being Lys-free) or that have Lys mutated into Arg on these individual sites (KyR, *y* = 6, 11, 27, 29, 33, 48, 63). As shown in Fig. 4h, K48-linked polyUb chains were most efficiently conjugated by MARCH2-mediated chains onto PGAM5, while MARCH2 appeared to conjugate K48R (Lys48-Arg)-linked polyUb chain poorly onto PGAM5 under MCM + H/R challenge (Supplementary Fig. S9b). Moreover, truncation mutants of MARCH2 were used to explore the nature of interacting domains. GST pull-down assay showed that deletion of amino acids 117–214 or 215–246 disengaged the binding of MARCH2 to PGAM5, suggesting crucial roles for TM and PDZ domains of MARCH2 in direct interaction with PGAM5 (Fig. 4i). Next, MS analysis was performed to map ubiquitination sites on PGAM5 and two lysine residues of PGAM5 capable of being ubiquitinated (K88 and K141) were yielded. Then, we generated *PGAM5* KO HL-1 cardiomyocyte cell line by CRISPR/Cas9-mediated genome editing technique to discern the ubiquitination sites of PGAM5. Remarkably, the combined mutants of K88R and K141R of PGAM5, PGAM5^K88,141R^, were slightly ubiquitinated by MARCH2 in MCM + H/R-challenged *PGAM5* KO cardiomyocytes (Fig. 4j). Furthermore, compared to WT PGAM5, the homeostatic level of PGAM5^K88,141R^ accumulated to a much higher level (Fig. 4k), most likely due to its longer half-life, as revealed by the pulse/chase experiment in the presence of CHX (Fig. 4l). Collectively, our findings suggest that MARCH2 promotes ubiquitination and degradation of PGAM5 by conjugating K48-linked polyUb onto K88 and K141 sites.

### MARCH2 suppresses PGAM5-dependent NLRP3 inflammasome activation following myocardial I/R

**Fig. 5:**
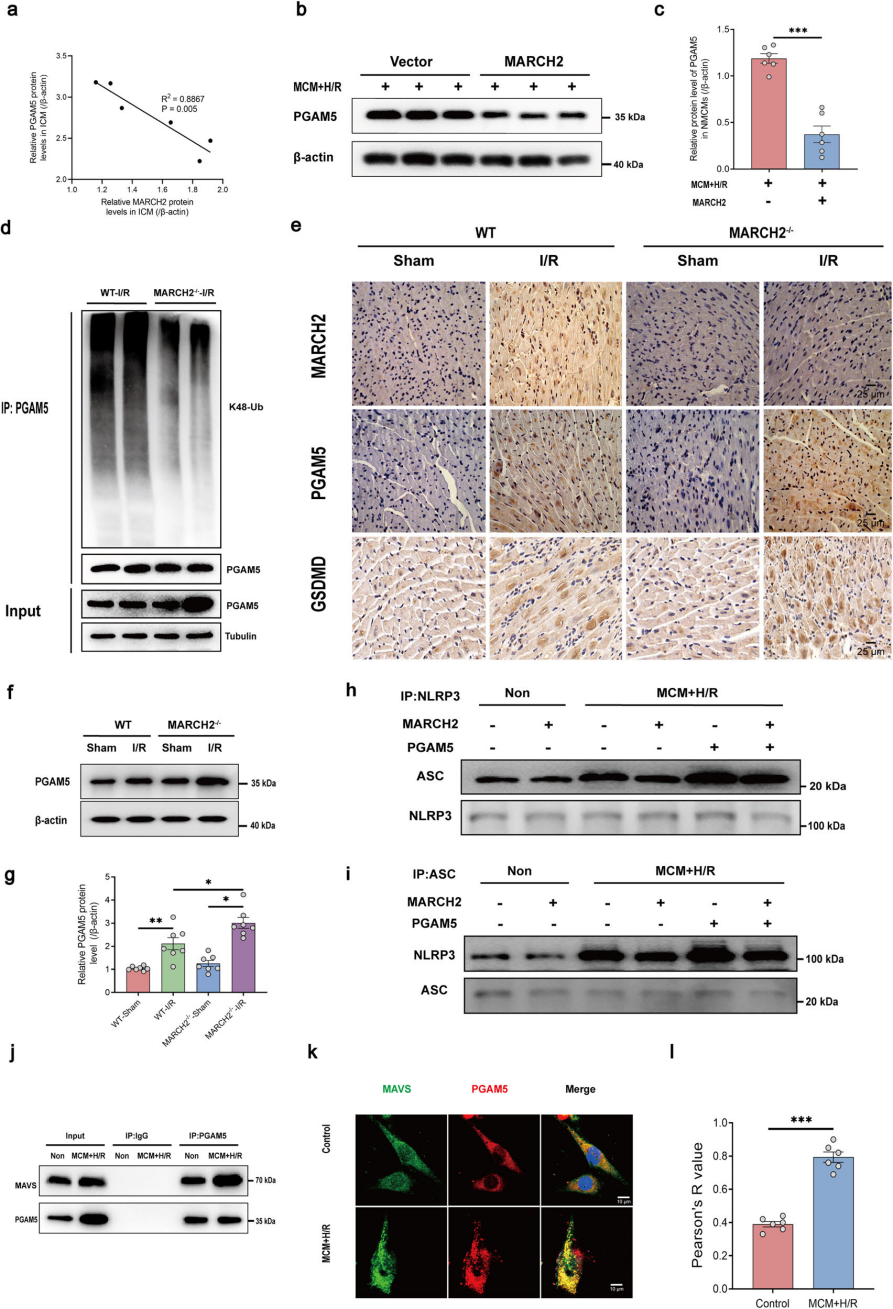
PGAM5 mediates the regulation of MARCH2 on NLRP3 inflammasome assembly following myocardial I/R. **a** Pearson correlation coefficients between MARCH2 and PGAM5 protein levels in ICM. **b**, **c** Representative western blotting (**b**) and quantitated analysis (**c**) of PGAM5 expression in NMCMs infected with vector or MARCH2 under MCM + H/R treatment. **d** Ubiquitination assays determining the ubiquitination of endogenous PGAM5 in the hearts of *MARCH2* KO and WT mice subjected to I/R injury. **e** Immunohistochemistry for MARCH2, PGAM5, and GSDMD in hearts of *MARCH2* KO and WT mice following I/R injury. **f**, **g** Representative western blotting image (**f**) and quantitated analysis (**g**) of PGAM5 in cardiac tissues of *MARCH2* KO and WT mice subjected to I/R (45 min/9 h). **h**, **i** Interaction between NLRP3 and ASC was examined by IP-western blotting assay in cardiomyocytes with MARCH2 or PGAM5 overexpression following MCM + H/R challenge. IP with NLRP3 antibody (**h**), IP with ASC antibody (**i**). **j** Interaction between PGAM5 and MAVS was examined by IP-western blotting assay in cardiomyocytes with or without MCM + H/R treatment. **k** Representative immunofluorescence images of MAVS and PGAM5 in HL-1 cells. **l** Colocalization analysis of PGAM5–MAVS. Pearson’s *R* value (no threshold) was calculated by ImageJ Fiji software. *n* = 6 images from 3 biological replicates. Data are shown as the means ± SEM. *P < 0.05, **P < 0.01, ***P < 0.001.

We then explored the potential roles of MARCH2-mediated PGAM5 degradation in cardiac I/R injury. Protein level of PGAM5 was negatively correlated with that of MARCH2 in patients with ICM (Fig. 5a). Protein level of PGAM5 was decreased in MARCH2-overexpressing NMCMs treated with MCM + H/R (Fig. 5b, c). Moreover, PGAM5 K48-linked ubiquitination was decreased in *MARCH2* KO hearts compared with WT mice after I/R injury (Fig. 5d). PGAM5 was also elevated in mouse hearts following I/R procedure, and such effect was further augmented by genetic ablation of *MARCH2* (Fig. 5e–g).

We next asked how MARCH2-mediated ubiquitin signaling affects PGAM5-mediated NLRP3 inflammasome. Co-IP assay with an anti-NLRP3 antibody in HL-1 cardiomyocytes indicated that MCM + H/R-elicited elevation of ASC was ablated by MARCH2 overexpression, which was counteracted by co-expression of PGAM5 (Fig. 5h, i). Moreover, the inhibition of MARCH2 on MCM + H/R-induced ASC oligomerization was mediated by PGAM5 (Supplementary Fig. S10).

### The formation of PGAM5–MAVS co-condensates under MCM + H/R treatment and PGAM5 promotes MAVS-dependent NLRP3 activation

Given the pivotal role of PGAM5 in regulating activation of NLRP3 inflammasome^21^, we went on to screen for potential PGAM5-interacting proteins starting from clues found in a public protein–protein interaction database (https://www.ebi.ac.uk/intact/). Indeed, PGAM5 was capable of interacting with MAVS, an adapter of NLRP3 inflammasome that is required for NLRP3 inflammasome activity^25^. We found that endogenous MAVS interacts with PGAM5 protein, as anti-PGAM5 enriched MAVS along with PGAM5 but the scramble IgG did not (Supplementary Fig. S11a). Co-IP assay was then performed in HL-1 cardiomyocytes expressing Flag-tagged MAVS and HA-PGAM5. Flag-MAVS was found in the HA-PGAM5 immune complexes (Supplementary Fig. S11b).

Next, we sought to evaluate potential role of PGAM5–MAVS interaction in response to MCM + H/R treatment. Ectopically expressed PGAM5 did not elicit notable change in the total cellular level of MAVS protein in H/R-challenged NMCMs (Supplementary Fig. S11c, d). A recent study reported that PGAM5 may serve as a scaffolding protein to allow the formation of PGAM5–MAVS complex for immunity activation^26^. Furthermore, PGAM5 and MAVS had both been shown to form puncta structures^26–28^. Therefore, we speculated that PGAM5 may serve as a scaffold protein co-condensed with the client protein MAVS to evoke MAVS-mediated activation of NLRP3 inflammasome^29^. We observed that MCM + H/R induced a more robust interaction between endogenous PGAM5 and MAVS in HL-1 cardiomyocytes (Fig. 5j) and NMCMs (Supplementary Fig. S12a). Likewise, MCM + H/R also promoted PGAM5–MAVS colocalization in the phase-separated condensates in HL-1 cardiomyocytes (Fig. 5k, l) and NMCMs (Supplementary Fig. S12b, c).

**Fig. 6:**
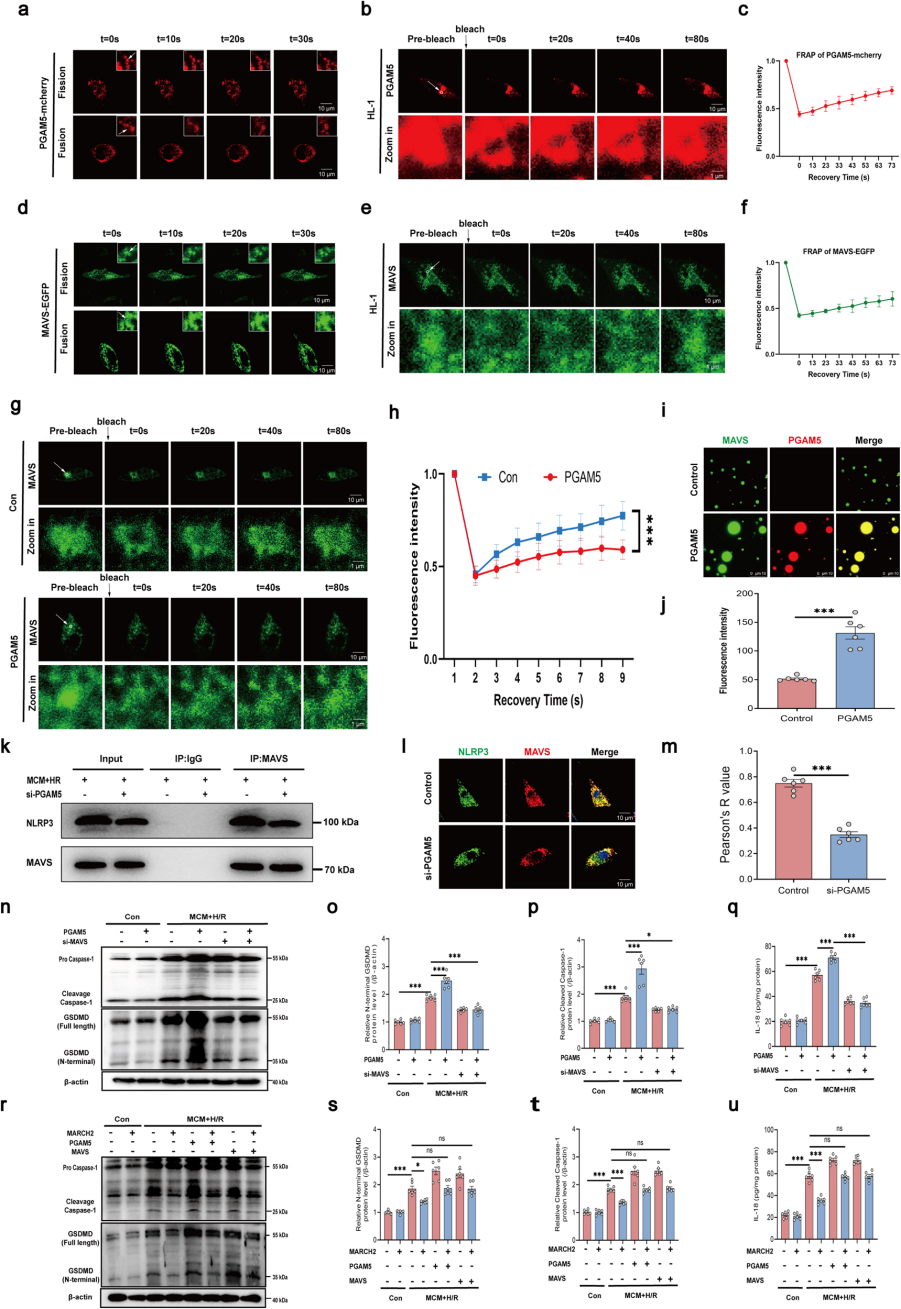
PGAM5–MAVS co-condensates form under MCM + H/R challenge and PGAM5 promotes MAVS-dependent NLRP3 activation. **a** Time-lapse images of HL-1 cells expressing PGAM5-mcherry. PGAM5 condensate fission and fusion is presented in the boxes. **b** FRAP analysis of PGAM5-mcherry condensates in HL-1 cells. **c** Quantification of FRAP in the bleached region of PGAM5-mcherry condensates, show as means ± SD (*n* = 6). **d** Time-lapse images of HL-1 cells expressing MAVS-EGFP. MAVS condensate fission and fusion is presented in the boxes. **e** FRAP analysis of MAVS-EGFP condensates in HL-1 cells. **f** Quantification of FRAP in the bleached region of PGAM5-mcherry condensates, show as means ± SD (*n* = 6). **g** FRAP analysis of MAVS-EGFP condensates in HL-1 cardiomyocytes without (upper) or with (lower) the presence of PGAM5. **h** Quantification of FRAP in the bleached region of MAVS-EGFP condensates with or without PGAM5, show as means ± SD (*n* = 6). **i** Representative fluorescent images of MAVS-EGFP droplets in the presence or absence of PGAM5. **j** Quantification of fluorescence intensity of MAVS-EGFP liquid droplets in **i**. **k** Interaction between NLRP3 and MAVS was examined by IP-western blotting assay in cardiomyocytes in the presence or absence of si-PGAM5 under MCM + H/R treatment. **l** Immunofluorescence of NLRP3 and MAVS in cardiomyocytes with or without PGAM5 knockdown under MCM + H/R treatment. **m** Colocalization analysis of NLRP3–MAVS. Pearson’s R value (no threshold) was calculated by ImageJ Fiji software. *n* = 6 images from 3 biological replicates. **n**–**p** Representative western blotting (**n**) and quantitated analyses of GSDMD (**o**) and caspase-1 (**p**) in cardiomyocytes infected with PGAM5 or si-MAVS under MCM + H/R treatment. **q** IL-18 release measured by ELISA in cardiomyocytes of the indicated groups underwent MCM + H/R treatment. **r**–**t** Representative western blotting (**r**) and quantitated analyses of GSDMD (**s**) and caspase-1 (**t**) in cardiomyocytes infected with MARCH2, PGAM5, or MAVS under MCM + H/R treatment. **u** IL-18 release measured by ELISA in cardiomyocytes of the indicated groups underwent MCM + H/R treatment. Data are shown as the means ± SEM. *P < 0.05, **P < 0.01, ***P < 0.001.

Our results indicated that PGAM5 condensates undergo fusion and fission in HL-1 cardiomyocytes (Fig. 6a) and HEK293T cells (Supplementary Fig. S13a) following MCM + H/R challenge, indicating liquid-like property of the PGAM5 condensates. Furthermore, fluorescence of PGAM5-mcherry also displayed a faster recovery following photobleaching under MCM + H/R challenge in HL-1 cardiomyocytes (Fig. 6b, c) and HEK293T cells (Supplementary Fig. S13b, c). Next, we reconstituted PGAM5 LLPS in vitro with purified full-length PGAM5-mcherry protein. As expected, PGAM5 droplets merged to grow with rising PGAM5 concentrations (Supplementary Fig. S13d), while the droplets disappeared with treatment of NaCl (Supplementary Fig. S13e). Moreover, MAVS-EGFP also formed condensates shown by droplet fission and fusion dynamically following MCM + H/R treatment in HL-1 cardiomyocytes (Fig. 6d) and HEK293T cells (Supplementary Fig. S13f). Upon photobleaching, MAVS-EGFP fluorescence rapidly recovered in HL-1 cardiomyocytes (Fig. 6e, f) and HEK293T cells (Supplementary Fig. S13g, h). Similarly, we reconstituted MAVS LLPS in vitro using purified full-length MAVS-EGFP protein. The formation of the MAVS-EGFP protein droplets was concentration-dependent (Supplementary Fig. S13i), and NaCl treatment abolished the formation of MAVS-EGFP droplets (Supplementary Fig. S13j). These results suggest that MAVS exhibits a dynamic liquid-like state under MCM + H/R treatment.

To test whether PGAM5 influences formation of MAVS condensate, fluorescence recovery after photobleaching (FRAP) assays were conducted in live HL-1 cardiomyocytes. The presence of PGAM5 prolonged MAVS-EGFP recovery process following photobleaching, suggesting that PGAM5–MAVS interaction might prompt the MAVS gel-like transition (Fig. 6g, h). Next, we examined whether PGAM5 induces MAVS phase separation in a cell-free condition using purified PGAM5-mcherry and MAVS-EGFP proteins. As expected, MAVS (30 μmol/L) formed more abundant and larger liquid droplets in the presence of PGAM5 (30 μmol/L) (Fig. 6i, j). To investigate whether PGAM5 can promote MAVS-mediated NLRP3 inflammasome activation, co-IP assay was performed in HL-1 cardiomyocytes in the presence or absence of PGAM5 following MCM + H/R treatment. Indeed, PGAM5 knockdown decreased binding between MAVS and NLRP3 in HL-1 cardiomyocytes (Fig. 6k) and NMCMs (Supplementary Fig. S14a). Furthermore, colocalization of NLRP3 and MAVS was significantly disrupted under PGAM5 knockdown in HL-1 cardiomyocytes (Fig. 6l, m) and NMCMs (Supplementary Fig. S14b, c). Of note, PGAM5 overexpression caused activation of NLRP3 inflammasome as shown by caspase-1 and GSDMD cleavage as well as IL-18 release in HL-1 cardiomyocytes following MCM + H/R treatment, the response to which was negated by MAVS knockdown (Fig. 6n–q; Supplementary Fig. S15a, b). To examine the formation of PGAM5–MAVS condensates in ischemic hearts in vivo, PGAM5 and MAVS were stained in sham and I/R mouse hearts and were found to exhibit an obvious condensation in I/R but not the sham hearts (Supplementary Fig. S16a, b). The number of PGMA5–MAVS condensates positively correlated with the PI-positive cardiomyocytes in hearts subjected to I/R injury (Supplementary Fig. S16c).

Moreover, MCM + H/R-evoked mitochondria-localization of inflammasome (colocalization of ASC and TOM20, Supplementary Fig. S17a), elevation of caspase-1 and GSDMD cleavage (Fig. 6r–t) as well as IL-18 release (Fig. 6u; Supplementary Fig. S17b, c) in HL-1 cardiomyocytes were blocked by MARCH2 overexpression, while PGAM5 or MAVS overexpression abrogated cardioprotective effects of MARCH2. Collectively, these results suggest a role of MARCH2 in inhibiting NLRP3 inflammasome activation in a PGAM5/MAVS-dependent manner.

### MARCH2 overexpression protects against myocardial I/R injury

**Fig. 7:**
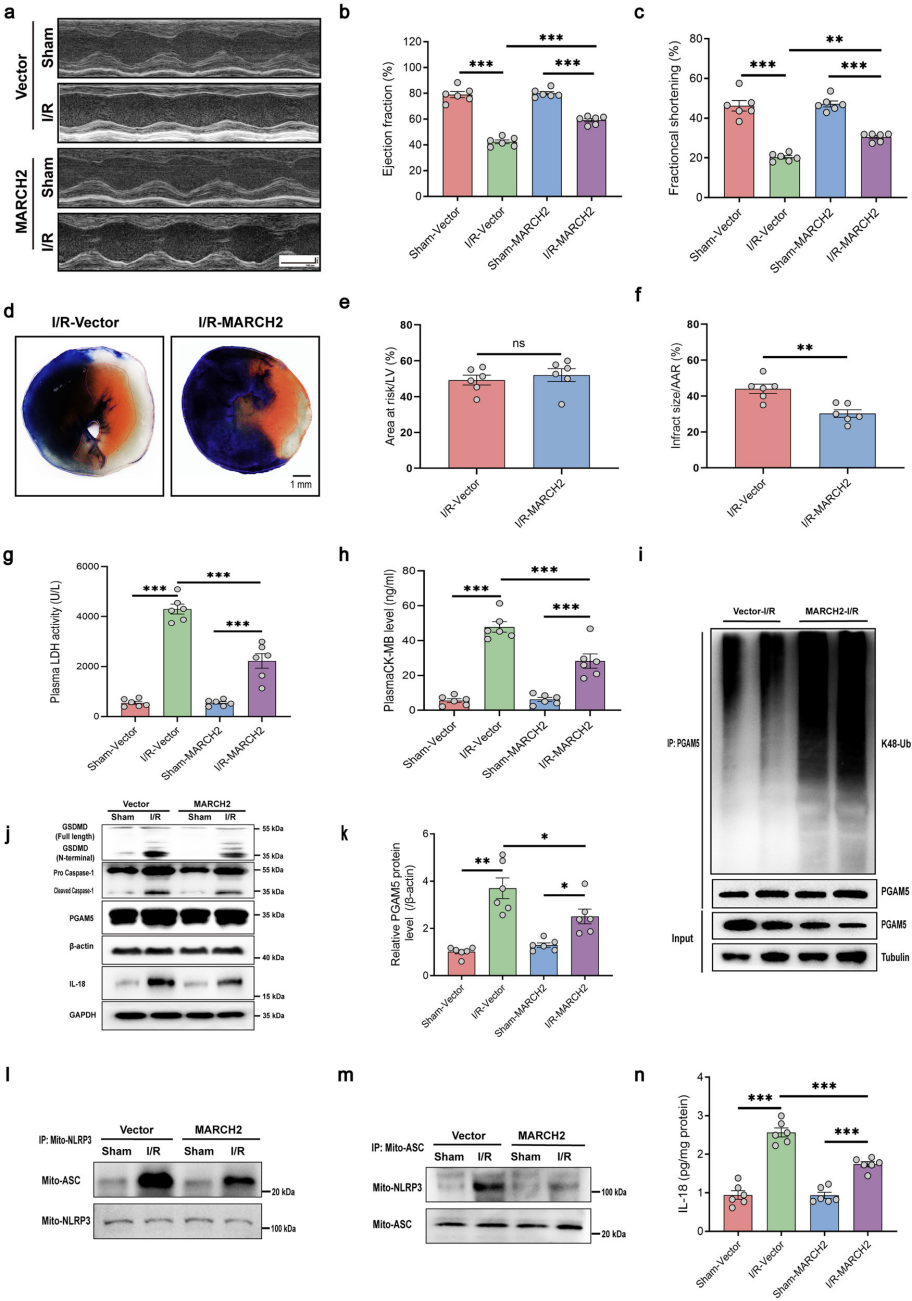
MARCH2 overexpression reduces I/R injury and preserves cardiac function via inhibition of PGAM5–MAVS-NLRP3 inflammasome pathway. **a** Representative M-mode recordings of echocardiography of WT mice injected with AAV9-cTnT-NC or AAV9-cTnT-MARCH2 (2 × 10^11^ V.g/mouse) for 3 weeks and then subjected to I/R (45 min/24 h). **b**, **c** Quantitative analysis of echocardiographic measurements performed in AAV9-cTnT-NC or AAV9-cTnT-MARCH2 mice subjected to I/R (45 min/24 h) injury (*n* = 6 mice per group). Left ventricular (LV) ejection fraction (EF, **b**) and fractional shortening (FS, **c**). **d** Representative images of heart sections by TTC/Evans Blue staining depicting infracted area. **e** Ratios of area at risk (AAR) to left ventricular (LV) area. **f** Infarct area normalized to AAR (*n* = 6 mice per group). **g**, **h** LDH activity and CK-MB level in AAV9-cTnT-NC or AAV9-cTnT-MARCH2 mice with or without myocardial I/R (45 min/24 h) (*n* = 6 mice per group). **i** Ubiquitination assays determining the ubiquitination of endogenous PGAM5 in the hearts of AAV9-cTnT-NC or AAV9-cTnT-MARCH2 mice subjected to I/R injury. **j** Representative western blotting analysis of PGAM5, caspase-1 (procaspase1; cleaved caspase-1), GSDMD (full-length; N-terminal), and IL-18 in hearts of AAV9-cTnT-NC or AAV9-cTnT-MARCH2 mice subjected to I/R (45 min/9 h). **k** Quantitated analysis of PGAM5 in hearts of AAV9-cTnT-NC or AAV9-cTnT-MARCH2 mice subjected to I/R (45 min/9 h). **l**, **m** Interaction between NLRP3 and ASC in mitochondria of cardiac tissues of AAV9-cTnT-NC or AAV9-cTnT-MARCH2 mice subjected to I/R was examined by IP-western blotting assay. **n** IL-18 release was measured by ELISA in cardiac tissues of AAV9-cTnT-NC or AAV9-cTnT-MARCH2 mice subjected to I/R. Data are shown as the means ± SEM. *P < 0.05, **P < 0.01, ***P < 0.001.

Given the observed role of MARCH2 in suppressing PGAM5/MAVS-mediated NLRP3 inflammasome activation, we explored therapeutic potential of MARCH2 for myocardial I/R injury. To this end, we constructed a viral vector to afford high MARCH2 expression in cardiomyocytes. Mice were delivered with AAV9-cTnT-MARCH2 3 weeks prior to I/R procedure. Notably, AAV9-cTnT-MARCH2 injection significantly elevated MARCH2 protein level in cardiomyocytes compared with AAV9-cTnT-Vector (Supplementary Fig. S18a, b). Remarkably, compared with AAV9-cTnT-Vector-injected mice, AAV9-cTnT-MARCH2-injected mice exhibited an overtly improved response to I/R injury, including improved cardiac function (Fig. 7a–c; Supplementary Fig. S18c–e) and decreased infarct size (Fig. 7d–f), as revealed by echocardiography and Evans blue/TTC double staining. As shown in Fig. 7g, h, levels of LDH and CK-MB, two markers for cardiac injury, were both less evident in the AAV9-cTnT-MARCH2 hearts compared to those received AAV9-cTnT-Vector. The PGAM5 K48-linked ubiquitination was increased (Fig. 7i) and PGAM5 protein level was decreased (Fig. 7j, k) in AAV9-cTnT-MARCH2 hearts compared to AAV9-cTnT-Vector hearts after I/R injury. Moreover, the intensified interaction between mitochondria-localized NLRP3 and ASC (Fig. 7l, m), caspase-1 and GSDMD cleavage (Fig. 7j), downstream proinflammatory IL-18 secretion after I/R (Fig. 7n) were reversed by AAV9-cTnT-MARCH2 injection. Altogether, MARCH2 manifests a previously unidentified protective property in cardiomyocytes against myocardial I/R injury, most likely through suppression of NLRP3 inflammation activation and cardiomyocyte pyroptosis.

### Cardiac PGAM5–MAVS signaling is essential for the protective effect of MARCH2 against myocardial I/R injury

Next, endogenous PGAM5 in cardiomyocytes was knocked down through RNA interference based on AAV9 viral vectors to test whether MARCH2-evoked cardioprotective effect is dependent upon PGAM5. WT and *MARCH2* KO mice were delivered with AAV9-cTnT-shPGAM5 3 weeks prior to I/R injury (Supplementary Fig. S19a). Evans blue/TTC double staining and echocardiography showed that cardiomyocyte-specific PGAM5 knockdown alleviated *MARCH2* KO-exacerbated effects as shown by infarct size (Supplementary Fig. S19a–c) and cardiac function (Supplementary Fig. S19d, e). Increases in LDH and CK-MB in *MARCH2* KO hearts were also restrained after cardiomyocyte-specific PGAM5 knockdown (Supplementary Fig. S19f, g). Consistently, increases in caspase-1 or GSDMD cleavage (Supplementary Fig. S19h–j) and IL-18 secretion (Supplementary Fig. S19k) in *MARCH2* KO hearts were decreased when PGAM5 was knocked down. Therefore, PGAM5/MAVS signaling is indispensable for the protective function of MARCH2 against cardiac I/R injury.

**Fig. 8:**
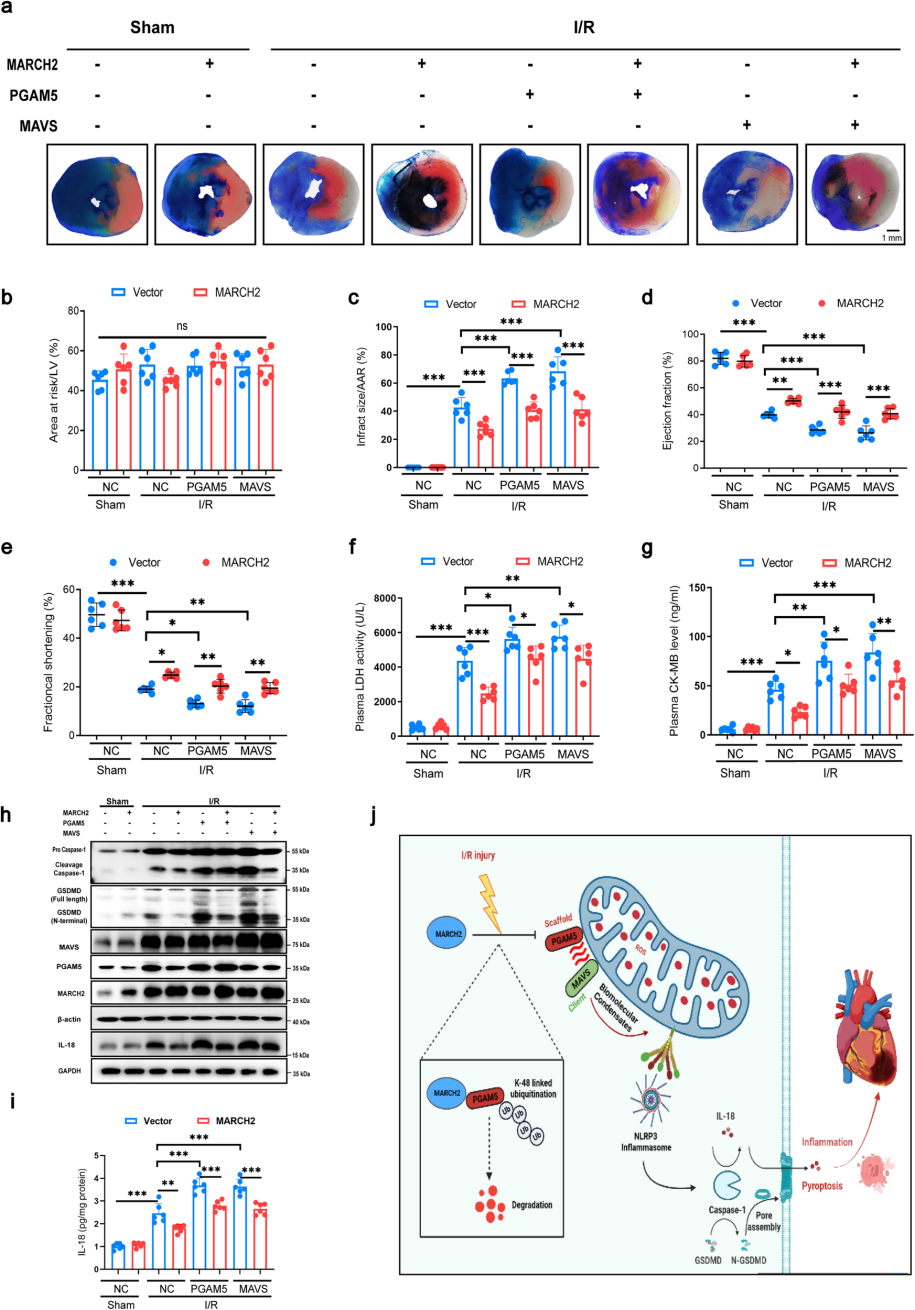
PGAM5/MAVS signaling pathway is essential for the regulation of MARCH2 in I/R injury. **a** Prior to I/R injury, WT mice were administrated with AAV9-cTnT-vector, AAV9-cTnT-MARCH2, AAV9-cTnT-PGAM5 or AAV9-cTnT-MAVS (2.0 × 10^11^ V.g/mouse) for 3 weeks by tail vein injection. TTC/Evans Blue staining is used to depict infracted area. **b** Ratios of area at risk (AAR) to left ventricular (LV) area. **c** Infarct area normalized to AAR. No statistical significance for Vector-I/R vs MARCH2-PGAM5-I/R, no statistical significance for Vector-I/R vs MARCH2-MAVS-I/R. **d**, **e** Echocardiographic assessment of ejection fraction (EF, **d**) and fractional shortening (FS, **e**) in the indicated groups. No statistical significance for Vector-I/R vs MARCH2-PGAM5-I/R, no statistical significance for Vector-I/R vs MARCH2-MAVS-I/R. **f**, **g** LDH activity (**f**) and CK-MB level (**g**) in mouse with and without myocardial I/R. No statistical significance for Vector-I/R vs MARCH2-PGAM5-I/R; no statistical significance for Vector-I/R vs MARCH2-MAVS-I/R. **h** Representative western blotting results of MARCH2, PGAM5, mito-MAVS, caspase-1 (procaspase1; cleaved caspase-1), GSDMD (full-length; N-terminal), and IL-18 in hearts of the indicated group mice subjected to I/R (45 min/9 h). **i** IL-18 release was measured by ELISA in cardiac tissues of the indicated group mice subjected to I/R (45 min/9 h). No statistical significance for Vector-I/R vs MARCH2-PGAM5-I/R, no statistical significance for Vector-I/R vs MARCH2-MAVS-I/R. **j** A working model for MARCH2 function in I/R injury. MARCH2 interacts with and promotes the degradation of PGAM5 by facilitating its K48-linked polyUb, thus inhibiting the activity of MAVS/NLRP3 inflammasome pathway and cardiomyocyte pyroptosis. Data are shown as the means ± SEM. *P < 0.05, **P < 0.01, ***P < 0.001.

To further pinpoint the essential role of PGAM5/MAVS signaling in regulating myocardial I/R injury by MARCH2, AAV9 viral vectors were employed to afford stable expression of MARCH2, PGAM5 and MAVS, respectively. Mice were delivered with AAV9-cTnT-MARCH2 with or without AAV9-cTnT-PGAM5 and AAV9-cTnT-MAVS for 3 weeks before I/R (Fig. 8a). Evans blue/TTC double staining showed that cardiomyocyte-specific MARCH2 overexpression in mice significantly reduced I/R-induced infarct size, which was blocked by cardiomyocyte-specific PGAM5 or MAVS overexpression (Fig. 8a–c). I/R-elicited dramatic myocardial injury as evidenced by overt decreases in EF and FS (Fig. 8d, e) and simultaneous elevation in cardiac injury markers, including serum LDH and CK-MB (Fig. 8f, g), all of which were off-set by MARCH2 overexpression. In contrast, the PGAM5 or MAVS overexpression caused the opposite effect. Furthermore, MARCH2 overexpression effectively suppressed I/R-evoked caspase-1 and GSDMD cleavage (Fig. 8h), reduced IL-18 secretion (Fig. 8i) and PI-positive cardiomyocytes (Supplementary Fig. S20), which were again abolished by co-expression of PGAM5 and MAVS. Taken together, these results demonstrated that MARCH2 protects against myocardial I/R injury, through suppression of NLRP3 inflammation assembly and pyroptosis, in a PGAM5/MAVS-dependent manner.

## Discussion

Our findings indicated a prominent cardioprotective role for MARCH2 against myocardial I/R injury, inflammasome activation, and cardiomyocyte pyroptosis. MARCH2 is upregulated in ICM patients and I/R-challenged mouse hearts. *MARCH2* ablation exacerbated I/R-evoked myocardial damage, as evidenced by infarct size, echocardiographic parameters, and cardiomyocyte mechanics. Our further analyses revealed that MARCH2 promotes degradation of PGAM5 by mediating its K48-linked polyUb, as well as mitigation of inflammatory response and pyroptotic cell death after I/R injury through a PGAM5/MAVS/NLRP3-dependent mechanism (Fig. 8j). In particular, PGAM5–MAVS condensates mediate activation of NLRP3 inflammasome, presumably acting as a PGAM5/MAVS/NLRP3 sponge and promoting caspase-1-dependent cleavage of GSDMD and IL-18. Moreover, ectopic expression of MARCH2 using AAV9-cTnT-MARCH2 effectively ameliorated I/R injury-evoked myocardial damages. In this context, MARCH2 is beneficial for I/R-elicited myocardial injury.

### MARCH2 protects against I/R-induced injury by reducing inflammatory response

MARCH2, a crucial regulator of innate immune system, curbs the activation of proinflammatory responses. *MARCH2* KO evokes excessive release of proinflammatory cytokines and correlates with a poor survival rate in response to bacterial infection and LPS-induced sepsis^20^. However, specific effect of MARCH2 in cardiovascular diseases remains unknown. Here, we reported that *MARCH2* deficiency does not affect normal cardiac function and structure but augments response to myocardial I/R injury. Cardiomyocyte-specific MARCH2 overexpression ameliorates I/R injury by curbing infarct size, improving cardiac function, and minimizing LDH release (Fig. 7a–h). Our data have thus revealed a previously unidentified protective role of MARCH2 in myocardial I/R injury. Mechanically, scRNA-seq results suggested that NOD-like receptor and cytokine-mediated signaling pathway genes were upregulated in cardiomyocytes of *MARCH2* KO mice compared to WT mice subjected to I/R, indicating a beneficial role for endogenous MARCH2.

Ample evidence has suggested a critical role for the activation of NLRP3 inflammasomes in myocardial damages and cardiomyocyte pyroptosis during myocardial I/R injury^1,11–13^. Upon I/R injury, mice with *NLRP3* deletion or knockdown with siRNA exhibited reduced infarct size and improved cardiac function compared with WT mice, suggesting a protective role of inhibiting NLRP3 in injury. Noticeably, MARCH2 was reported for its role in negatively regulating NF-κB and type I interferon signalings^20^. Our present study demonstrated that MARCH2 restrains NLRP3 inflammasome complex assembly and subsequent myocardial pyroptosis. *MARCH2* ablation augments I/R-induced elevation in levels of NLRP3, ASC, cleaved caspase-1, N-terminal GSDMD, and IL-18. Consistently, overexpression of MARCH2 blocks activation of NLRP3 inflammasome and cardiomyocyte pyroptosis, suggesting an essential role for MARCH2 in the regulation of inflammasome activation and myocardial damage (Fig. 7j–n).

### MARCH2 promotes ubiquitination and degradation of PGAM5 by catalyzing K48-linked polyUb

Protein ubiquitination, a versatile post-translational modification, plays critical roles in cardiovascular system development, whose aberrances either feature or underlie a variety of cardiovascular diseases, including myocardial I/R injury^30,31^. The functional diversity of ubiquitination stems from structurally intricate polyUb chains, in which a single ubiquitin chain can be further ubiquitinated to form branched chains^32–35^. K48-linked chains are the prominent linkage form in cells that may target substrate proteins for the 26 S proteasomal degradation^36^. Previously, the ubiquitin modification of PGAM5 was reportedly catalyzed by E3 ligase RNF5, likely through K48-linked polyUb chains, which is known to inhibit the inflammatory responses in hepatic I/R injury^37^. In this study, we presented an array of data showing that the E3 Ub ligase MARCH2, which is abundantly expressed in cardiomyocytes and becomes upregulated during myocardial I/R injury, interacts explicitly with PGAM5, where the TM and PDZ domains of MARCH2 were attributed as the binding sites. Furthermore, MARCH2 mediates K48-linked polyUb modifications of PGAM5 under H/R challenge, targeting it to the proteasome for degradation. Interestingly, mutation of Lys residues 88 and 144 of PGAM5 led to decreased degradation of PGAM5 protein. Therefore, these Lys residues may serve as sites on PGAM5 to receive MARCH2-mediated ubiquitin signaling.

### MARCH2 inhibits NLRP3 inflammasome activation through the PGAM5/MAVS/NLRP3 pathway

In this study, PGAM5 was found to interact with MARCH2, and MARCH2 conjugates K48-linked polyUb chains onto PGAM5, thus accelerating PGAM5 degradation (Fig. 4). Moreover, PGAM5 negatively correlated with MARCH2 in ICM and mediated regulation of MARCH2 on the MAVS/NLRP3 axis in myocardial I/R injury (Fig. 5). Our results indicate a crucial role for MARCH2-mediated K48-linked PGAM5 polyUb in myocardial I/R injury.

PGAM5 is a serine/threonine-protein phosphatase belonging to the PGAM family. It localizes in outer mitochondrial membrane and participates in a wide range of biological processes, including Wnt/β-catenin signaling^38,39^, redox regulation^40^, immune response^41^, and mitochondrial quality control^42,43^. Besides, PGAM5 functions at the converging point of several cell death pathways, including necrotic cell death^22,44,45^ and ROS-induced cell death^46^. Recently, it was reported that PGAM5 is dispensable for necroptosis although it offers a pivotal role in inflammasome activation and IL-1β release^21^. Our data suggested that protein level of PGAM5 was elevated in hearts of mice subjected to I/R injury. Cardiac-specific overexpression of PGAM5 exacerbated I/R-elicited infarct size, cardiac dysfunction, NLRP3 inflammasome assembly, and myocardial pyroptosis, indicating PGAM5-aggravated cardiac dysfunction and inflammation responses following I/R injury (Fig. 8). Moreover, inhibition of PGAM5 could also reduce necroptosis in rat hearts following I/R injury through suppression of dynamin-related protein 1^47^, suggesting a key role for PGAM5-regulated other forms of cell death.

The optimal function of NLRP3 inflammasome is achieved through its recruitment to mitochondria by MAVS, a mitochondrial outer membrane-localized, antiviral signaling protein, and plays a pivotal role in innate immune system^25^. Here, we found that cardiac-specific overexpression of MAVS aggravates the deleterious effects of NLRP3 inflammasome activation and cardiac damages upon myocardial I/R injury, suggesting a deleterious effect of MAVS upregulation during cardiac injury^48^.

Biomolecular condensate formation regulated by LLPS in biology is now considered an essential mechanism to orchestrate complex cellular processes by concentrating certain molecules or excluding unwanted others^49^. Aberrant LLPS contributes to inflammasome activation,^50^ innate immunity^51^, and pathogenesis of arterial stiffening^52^. However, the potential roles of biomolecular condensation in cardiac diseases remain poorly understood. Here, we demonstrated that PGAM5 and MAVS are capable of undergoing LLPS in cardiomyocytes following MCM + H/R treatment. The compositions of biocondensates include scaffolds and clients. Scaffold proteins are elementary and essential for condensate formation, and the partition into condensates of client proteins is regulated by the scaffolds^29^. There is little information concerning how biocondensates are involved in cardiac physiology and pathophysiology. We found that PGAM5 functions as a scaffold protein that co-condenses with the client protein MAVS. It presumably acts like a PGAM5/MAVS/NLRP3 sponge to promote NLRP3 inflammasome activation. Therefore, targeting PGAM5 for proteasomal degradation by MARCH2-mediated proteolytic ubiquitin signaling serves as an endogenous brake on MAVS-NLRP3 inflammasome activation to alleviate myocardial injury during I/R stress. Therapeutic upregulation or activation of MARCH2 thus provides a novel avenue to combat these related diseases.

In summary, findings from our study not only established the E3 ligase MARCH2 as a novel cardioprotective molecule against myocardial I/R injury, but also identified a previously unknown mechanism that negatively regulates PGAM5/MAVS/NLRP3 inflammasome signaling and pyroptosis to maintain homeostasis and normal function of mammalian hearts. Targeting MARCH2 may thus represent a novel opportunity to battle against myocardial injuries.

## Materials and methods

### Human donors

This study was performed according to the ethical guidelines of the Declaration of Helsinki. Experimental procedures were approved by the institutional review board of the Sun Yat-Sen Memorial Hospital, Sun Yat-Sen University (SYSEC-KY-KS-2021-070). Patients who have undergone heart transplant surgery due to ischemic heart disease were recruited in the study. Control donor hearts were obtained from participants who died in car accidents. Written informed consent was obtained from patients.

### Animals

All animal procedures were approved by the institutional animal care and use committee of Zhongshan Hospital, Fudan University. Experiments were performed according to the institutional guidelines.

WT C57BL/6 J mice were purchased from Cyagen Biosciences Inc. (Guangzhou, China). *MARCH2* KO mice were generated using CRISPR/Cas9 system. SgRNA targeting the sequence ACGGGATGGCCGGCTGCTC in exon 2 of mouse *MARCH2* and Cas9 mRNA were injected into one-cell embryos for producing the offspring. The primers employed for *MARCH2* genotyping were 5’-CTGCTGTTGTGACTCACTAATTTTG-3’ and 5’-CCAATGAGCCATTCCTCCAACATC-3’. Genotype was further verified by Sanger sequencing. The founder mouse with a 19 bp deletion in exon 2 of *MARCH2* was utilized for our experiments (Supplementary Fig. 2a). Only F3 and beyond were used in this study.

### Myocardial I/R procedure

Male mice aged 7–8 weeks were anesthetized with 2% isoflurane with 100% O_2_ ventilation at 2 L/min. Myocardial ischemia was induced using left anterior descending coronary artery (LAD) ligation by knotting a silk suture (6–0) around LAD. After 45 min of occlusion, the slipknot was released to allow a reperfusion period ranging 3–24 h. Sham surgery was performed by making a left thoracic incision to expose the pericardium except leaving the LAD intact. After reperfusion, blood samples were collected for LDH and CK-MB measurements.

### Measurements of infarct area and area at risk

24 h after I/R injury, LAD artery was reoccluded and retied at the same ligation site. Evans blue dye (0.2 mL 1%) was injected into aorta to demarcate the ischemic AAR. Then, the heart was excised and subsequently cross-sectioned into 1-mm slices, which were incubated in 1% 2,3,5-triphenyltetrazolium chloride (TTC) solution for 10 min at 37 °C to delineate infarct size. The percentages of infarcted area (pale), AAR (brick red), and total left ventricular (LV) area of each myocardial sections were measured using Image J software, and the values were averaged.

### Echocardiography

Transthoracic echocardiography was performed in mice using a high-resolution micro-imaging system (Visual Sonics Vevo 2100, Toronto, ON, Canada) to evaluate cardiac function and structure. Standard two-dimensional M-mode measurements was performed at 24 h after I/R surgery on mice anesthetized with 2% isoflurane. Measurements of end systole and end diastole LV internal diameters, LV anterior/posterior wall thickness, and interventricular septum were performed using M-mode recordings.

### Cardiomyocyte isolation and cell culture

NMCMs were isolated according to the previous report^53^ with modifications. In brief, hearts from 1-day-old mice were minced and digested with collagenase I (Worthington) for 3 min at 37 °C. The collected cells were incubated in fresh culture medium (DMEM containing 20% fetal calf serum (FBS)) at 37 °C and 5% CO_2_ for 90 min to separate cardiac fibroblasts from cardiomyocytes.

Adult cardiomyocytes were enzymatically isolated from adult mouse hearts according to a previous report^3^. Adult cardiomyocytes were plated onto laminin (5 μg/mL, Sigma, St. Louis, USA)-precoated culture plates.

HEK293T and HL-1 murine cardiomyocyte, Raw264.7 murine macrophages were obtained from ATCC and cultured in Dulbecco’s modified Eagle’s medium (DMEM) containing 10% FBS, and penicillin/streptomycin under a humidified atmosphere containing 5% CO_2_ at 37 °C.

MCM was obtained from the medium of Raw264.7 macrophages treated with LPS (200 ng/mL, Sigma) for 6 h and NLRP3 activator ATP (2 nM, Sigma) for 30 min. To mimic the H/R injury and inflammatory environment, HL-1 cardiomyocytes and NMCM cardiomyocytes were treated with MCM in H/R (3 h/6 h) atmosphere.

### Cell shortening/re-lengthening

Mechanical properties of cardiomyocytes were assessed using a Soft-edge MyoCam system (IonOptix Corporation, Milton, MA, USA) equipped with an IX-70 Olympus inverted microscope. Single cardiomyocytes were electrically stimulated at 0.5 Hz in a contractile buffer to detect these properties.

### Plasmid, small RNA interference, and adenovirus

*MARCH2*, *PGAM5*, *MAVS,* and *Flag-Ub* were amplified from HEK293T cDNA through standard PCR techniques, and then inserted into pcDNA3.1 (+) vectors. Point mutations of the indicated plasmids of ubiquitin chain were introduced through site-directed mutagenesis.

*PGAM5-mcherry*, *MAVS-EGFP*, and the selective siRNAs and negative controls were designed and synthesized by Jiangsu Gencefe Biotechnology Co., Ltd. (Jiangsu, China). Control siRNA was employed as a negative control under similar conditions. siRNAs used in our experiment were: mouse *MARCH2* siRNA sequence: 5′-GCCACCUCAAUAUGUAGCACATT-3′; mouse *MAVS* siRNA sequence: 5′-CCACCUUGAUGCCUGUGAATT-3′. Plasmid or siRNA transfection was conducted using Lipofectamine 3000 reagent (Thermo Fisher Scientific, USA).

Adenovirus expressing *MARCH2*, *shMARCH2*, *PGAM5*, *MAVS* were conducted by the OBiO Technology Corp., Ltd. (Shanghai, China). For *MARCH2*, *PGAM5,* or *MAVS* overexpression in mice, the entire coding region of the mouse *MARCH2*, *PGAM5,* or *MAVS* cDNA was subcloned into the *pHBAAV9-cTNT-MCS* vector downstream of the cardiac troponin-T promoter. Mice were given adenovirus (2.0 × 10^11^ V.g /mouse) using tail intravenous injection. Cardiac-restricted *MARCH2*, *PGAM5,* or *MAVS* overexpression was successfully induced 3 weeks after AAV9 transfection in adult C57BL/6 J mice. The corresponding control mice received *pHBAAV9-cTNT*-Vector injections at the same dose respectively. For PGAM5 knockdown, AAV9 harboring *PGAM5* shRNA (*pAAV-cTNT-miR30* shRNA (*PGAM5*)) was constructed.

### Generation of PGAM5 knockout cell lines

*PGAM5*^*–/–*^ HL-1 cardiomyocyte cell line was generated by CRISPR/Cas9-mediated genome editing technique as previously described. The sgRNAs targeting *PGAM5* were designed using online tool (http://crispr.mit.edu/). The sgRNA-expressing plasmids were transfected into HL-1 cells with Lipofectamine 2000 (Invitrogen, Carlsbad, CA, USA) for 24 h, and selected with puromycin (Sigma) for single colonies. Single-cell colonies were picked, amplified, and confirmed by immunoblotting analysis. We then extracted genomic DNAs, amplified the specific target sequences, and performed Sanger sequencing to verify the aimed edits in the genomes of the HL-1 cell line.

### Western blotting assay

Total protein was extracted from heart tissues or cells. Equal amounts of protein (generally 25 or 50 μg) were separated by SDS-PAGE gels and subsequently transferred to a nitrocellulose membrane. After blocking with 5% bovine serum albumin (BSA), the membranes were incubated with the indicated primary antibodies at 4 °C overnight.

Antibodies used were anti-MARCH2 (1:1000, Abcam, Ab123136), anti-PGAM5 (1:1000, Abcam, ab126534), anti-MAVS (1:1000, Cell Signaling Technology, #83000), anti-GSDMD (1:1000, Abcam, ab209845), anti-NLRP3 (1:1000, Cell Signaling Technology, #15101), anti-Caspase-1 (1:1000, Proteintech, 22915-1-AP), anti-ASC (1:1000, Cell Signaling Technology, #67824), anti-VDAC1 (1:1000, Abcam, ab154856), anti-VDAC2 (1:1000, Proteintech, 11663-1-AP), anti-HSP90 (1:1000, Proteintech, 11405-1-AP), anti-MFN2 (1:1000, Proteintech, 12186-1-AP), anti-MCU (1:1000, Abcam, ab219827), anti-Ubiquitin (K48) (1:1000, Abcam, ab140601), Anti-Flag (1:1000, Cell Signaling Technology, #14793), Anti-HA (1:1000, Cell Signaling Technology, #2367), Anti-Myc (1:1000, Cell Signaling Technology, #2276). Horseradish-peroxidase (HRP)-conjugated β-actin (1:1000, Proteintech, HRP-60008), HRP-conjugated α-Tubulin (1:1000, Proteintech, HRP-66031).

After washed with TBST for three times, membranes were incubated with HRP-conjugated secondary antibodies at room temperature for 1 h. Bands were scanned by enhanced chemiluminescence luminal reagents (Bio-Rad Laboratories). Gray value was qualified by Image Lab (Bio-Rad, USA).

For chemical cross-linkage of ASC oligomerization, the Triton X-100 insoluble pellet was re-suspended in PBS buffer and chemically crosslinked with 4 mM disuccinimidyl suberate (DSS) at room temperature for 30 min.

### RT-qPCR

Total RNA was extracted from heart tissues or cells using Trizol Reagent (Invitrogen). First-strand cDNA was generated from total RNA using the Prime Script™ RT Master Mix Kit (Takara, Tokyo, Japan). RT-qPCR was performed in 25 μL reactions including 0.4 μmol/L primers, 50 ng cDNA, and 12.5 μL TB Green Premix Ex Taq II (Takara) using the CFX96 Real-Time PCR Detection System (Bio-Rad Laboratories). The expression level of targeted genes was normalized to that of *belta-actin*, which was regarded as an endogenous internal control. Primer sequences were as follows: mouse *MARCH2*: forward primer 5′-AGGGCTCAGAGGTAGTAGACA-3′, reverse primer 5′-AAATATGTTCACACGGGATCACA-3′; mouse *belta-actin*: forward primer 5′-GGCTGTATTCCCCTCCATCG-3′, reverse primer 5′-CCAGTTGGTAACAATGCCATGT-3′; mouse *PGAM5*: forward primer 5′-ATCTGGAGAAGACGAGTTGACA-3′, reverse primer 5′-CCTGTTCCCGACCTAATGGT-3′.

### Cell model of H/R injury

Cardiomyocytes were placed in an “ischemic buffer” (118 mM NaCl, 24 mM NaHCO_3_, 1.0 mM NaH_2_PO_4_, 2.5 mM CaCl_2_-2H_2_O, 1.2 mM MgCl_2_, 20 mM sodium lactate, 16 mM KCl, and 10 mM 2-deoxyglucose, pH adjusted to 6.2) and transferred to a controlled hypoxic chamber (1% O_2_, 94% N_2_, 5% CO_2_) for 30 min. Then cardiomyocytes were placed back in a normal humidified atmosphere containing 5% CO_2_ at 37 °C and replaced with a culture medium for reoxygenation.

### Immunofluorescence staining and live cell imaging

Cells grown on confocal dish were washed with PBS, then fixed with 4% paraformaldehyde for 15 min, and permeated with 0.1% Triton X-100 plus 1% BSA for 1 h at room temperature. Subsequently, primary antibodies against Flag (Cell Signaling Technology, Cat. #8146), HA (Cell Signaling Technology, #3724), ASC (Santa Cruz, #SC-514414), TOM20 (Abcam, #ab186735) were used to label cellular proteins at 4 °C overnight. After washed with PBS for three times, cells were then stained with a fluorescent second antibody at 37 °C for 1 h. DNA was stained with DAPI. The fluorescence signals of fixed cells and live cells were acquired using a confocal microscopy (Leica TCS SP8).

Quantification of fluorescence intensity was performed with ImageJ software. Statistic data were acquired from ~40–100 cells from 6 random fields from at least three independent experiments. ImageJ Fiji software (Analyze-colocalization-coloc2) was used to calculate the Pearson colocalization coefficient. Statistic data were obtained from 6 random fields from at least 3 independent experiments.

### ELISA

Levels of CK-MB and LDH were determined as markers for cardiac injury. Levels of CK-MB and LDH were measured using CK-MB assay and LDH assay kits (Nanjing Jiancheng, Nanjing, China) according to the manufacturer’s instructions.

### Mitochondrial purification

Mitochondria were isolated with a mitochondrial isolation Kit (Abcam, Cambridge, USA). Briefly, cells were harvested and homogenized in isolation buffer. Following centrifugation at 1000 × *g* for 10 min, the supernatant was collected and was then centrifuged at 12,000 × *g* for 15 min at 4 °C, yielding enriched mitochondria in pellets. Mitochondrial pellets were re-suspended using a radioimmunoprecipitation assay (RIPA) buffer containing protease inhibitor cocktail. Cardiac tissues (30–50 mg) were washed in wash buffer and homogenized in isolation buffer. Then homogenized tissue was centrifuged at 1000× *g* for 10 min, supernatants were collected. After centrifuged at 12,000× *g* for 15 min, pellets were resuspend using RIPA buffer and were quantified using the Bradford method.

### FRAP assay

Cells were grown on glass-bottom dishes until they reached the appropriate density. Cells were then subjected to MCM + H/R treatment. GFP or mcherry signals in regions of interest (ROI) were fully photobleached by using a 488-nm laser on a Leica SP8 confocal microscope. Fluorescence intensity of ROI between pre-bleached and at the start of recovery after bleaching was recorded by microscope. FRAP data were analyzed using GraphPad Prism 8.4.

### In vitro droplet assay

Purified MAVS-EGFP and PGAM5-mcherry proteins were diluted to the indicated concentrations with a phase separation buffer containing 20 mM Tris-HCl (pH = 7.5), 75 mmol/L KCl, 10% BSA, 5% PEG, and 1 mmol/L DTT, and the mixtures were incubated at 37 °C for 10 min to induce phase separation.

### Protein purification

Plasmid of pGEX-4T-1-GST or pGEX-4T-1-GST-PGAM5, pGEX-4T-1-GST-MARCH2-truncated proteins, pGEX-4T-1-MAVS-EGFP was expressed in *Escherichia coli* strain BL21 competent cells and induced with IPTG at 16 °C for 12 h. Bacteria were collected by centrifugation and lysed by sonication. Lysate was immobilized on glutathione-agarose beads (GE Healthcare) following the manufacturer’s protocols; pET28a-His-MARCH2, pET28a-His-PGAM5, or pET28a-His-PGAM5-mcherry proteins were purified with Ni-NTA agarose beads (Qiagen) per the manufacturer’s instruction. The pull-down assays of other proteins followed the same procedures.

### IP assay

Cells were lysed with 500 μL NP-40 lysis buffer (20 mM Tris-HCl pH 7.4, 1% NP-40, 135 mM NaCl, 10% glycerol, 0.2 mM PMSF, phosphatase inhibitors, and protease inhibitor). Lysates were collected and subjected to centrifugation. After centrifugation, 50 μL of supernatant was aliquoted for input. The remained supernatant was immunoprecipitated with the indicated primary antibodies and protein A/G Agarose beads (Santa Cruz Biotechnology) at 4 °C. Anti-mouse IgG (Santa Cruz Biotechnology, #sc2025) was used as a control. The IP complexes were washed four times with lysis buffer to remove nonspecifically bound protein. Both input and immunoprecipitates were examined by immunoblotting.

### IP/MS

Following transfection with Flag-tagged MARCH2 in HEK293T cells, IP was performed using an anti-Flag primary antibody. Precipitates were separated using SDS-PAGE gels and were subsequently stained with Coomassie blue. Bands that differed between the Flag-MARCH2 and control groups were analyzed using liquid chromatograph-electrospray ionization-mass spectrometry (Shanghai Biotechnology, Shanghai, China).

### Mass spectrometry analysis to map ubiquitylation modification sites on substrates

Procedures for MS analysis were as previously described^34^. In brief, the protein pellet was concentrated in 8 M urea, 100 mM Tris-Cl (pH 8.5), followed by TCEP reduction, NEM alkylation, and trypsin digestion. Peptides were separated and analyzed by the EASY-nLC system (Thermo Fisher) and the Q Exactive mass spectrometer (Thermo Fisher), respectively. Ubiquitylation modification sites were determined with Thermo Proteome Discoverer 2.1 (Thermo Fisher) and searched in Uniprot Human database (http://www.uniprot.org/).

### PI staining

Mice were injected with PI (Sigma, 10 mg/kg, i.p.) to label necrotic cells following I/R surgery. Briefly, hearts were quickly removed and washed with PBS, embedded in OCT, frozen in liquid nitrogen, and cut into 5-μm sections. Frozen sections were then stained with DAPI. The immunofluorescence staining was conducted as described above.

### Ubiquitylation assays

For in vivo PGAM5 ubiquitylation assay, HEK293T and HL-1 cells were transfected with Myc-MARCH2, HA-PGAM5, and Flag-ubiquitin using Lipofectamine 3000. 24 h later, whole cells were lysed with lysis buffer (50 mM Tris-HCl, pH 7.4, 150 mM NaCl, 1% Triton X-100, 5 mM EDTA, 0.1% SDS, 0.5% sodium deoxycholate, with phosphatase and protease inhibitors). The lysates were centrifuged to obtain supernatant proteins and incubated with anti-Flag antibody (Cell Signaling Technology) for 3 h and protein A/G agarose beads (Santa Cruz Biotechnology) for 3 h at 4 °C. Then the immune complex was washed extensively for four times with lysis buffer and boiled with SDS-PAGE sample buffer for 10 min. Ubiquitination was analyzed by immunoblotting with anti-Flag monoclonal antibody (Cell Signaling Technology).

### ScRNA-seq and data analyses

WT male mice and *MARCH2* KO male mice aged 7–8 weeks suffering sham or I/R injury were used for scRNA-seq (3 mice/group). ScRNA-seq was conducted by Singleron Biotechnologies (Shanghai, China). In brief, cells were isolated from heart tissues using GEXSCOPE Nuclear Extraction Buffer (adding RNase inhibitor and DTT before use, the final concentrations of the two are 0.2 U/µL, 1 mM, respectively). Single-cell suspensions at a concentration of 1 × 10^5^ cells/mL in PBS were prepared. Single-cell suspensions were then loaded onto microfluidic devices and scRNA-seq libraries were constructed according to Singleron GEXSCOPE protocol using GEXSCOPE Single-Cell RNA Library Kit (Singleron Biotechnologies), which included cell lysis, mRNA trapping, labeling cells (barcode) and mRNA (UMI), mRNA reverse transcription, cDNA amplification, and finally cDNA fragmentation. Individual libraries were diluted to 4 nM and pooled for sequencing. Pools were sequenced on an Illumina HiSeq X with 150 bp paired-end reads. Raw reads were processed with fastQC and fastp to remove low quality reads. Poly-A tails and adaptor sequences were removed by cutadapt. Reads were mapped to the reference genome GRCh38 (ensembl version 92 annotation) using STAR. Gene counts and UMI counts were acquired by featureCounts software. Cells were filtered by gene counts below 200 and the top 2% gene counts and the top 2% UMI counts. Cells with over 20% mitochondrial content were removed. After filtering, 178,274 cells were retained for the downstream analyses, with on average 1794 genes and 3808 UMIs per cell. Expression matrix files for subsequent analyses were generated based on gene counts and UMI counts. FindMarker (Seurat) was used for DEG analysis. To identify the genes that may participate in the pathogenesis of myocardial I/R injury, we screened candidate genes from the scRNA-seq of WT-sham group and WT-I/R group adopting the following strategy: (1) upregulated DEGs (lnx-FoldChange > 0.25, adjusted *P* values < 0.05) of the comparison were partitioned; (2) downregulated DEGs (lnx-FoldChange2 < –0.25, adjusted *P* values < 0.05) of the comparison were partitioned; (3) the filtered upregulated DEGs and downregulated DEGs were then intersected with the E3 Ub ligase database.

### Statistical analysis

For statistical analysis, all quantitative data are presented as means ± SEM of at least three independent experiments. Data distribution was first examined using the Shapiro–Wilk normality test. Statistical differences were performed using a two-tailed unpaired Student’s *t*-test for comparison between two groups. Statistical differences among three or more groups were determined by one-way ANOVA or two-way ANOVA (for two-factor levels), followed by Bonferroni’s multiple comparison test. *P < 0.05, **P < 0.01, ***P < 0.001 were considered significant. Statistical analyses were carried out using GraphPad prism 8.0 and SPSS Version 19.0.

### Supplementary information

Supplementary information

## Data Availability

Raw scRNA-seq data have been deposited in the National Omics Data Encyclopedia (https://www.biosino.org/node/search) under the accession number: OEP004577.
