# Supplementary material for: The E3 ubiquitin ligase MARCH2 protects against myocardial ischemia-reperfusion injury through inhibiting pyroptosis via negative regulation of PGAM5/MAVS/NLRP3 axis
Source: Cell Discov. 2024 Feb 27;10:24. doi: 10.1038/s41421-023-00622-3 (PMC10897310; doi:10.1038/s41421-023-00622-3)
Supplement: Supplementary file 1 — Supplementary information [file 41421_2023_622_MOESM1_ESM.pdf]

Supplementary Material

The E3 ubiquitin ligase MARCH2 protects against myocardial ischemia-reperfusion injury through inhibiting pyroptosis via negative regulation of PGAM5/MAVS/NLRP3 Axis

Shuolin Liu[1], Yaguang Bi[1], Tianting Han[2,3], Yiran E. Li[1], Qihang Wang[2,3], Ne Natalie Wu[1], Chenguo Xu[2], Junbo Ge[1*], Ronggui Hu[2,3,4,5*], Yingmei Zhang[1*]

1. Department of Cardiology, Zhongshan Hospital, Fudan University, Shanghai Institute of Cardiovascular Diseases, National Clinical Research Center for Interventional Medicine, Key Laboratory of Viral Heart Diseases, National Health Commission. Key Laboratory of Viral Heart Diseases, Chinese Academy of Medical Sciences, Shanghai, China.
2. College of Basic Medicine, Shanghai Medical College, Fudan University, Shanghai China.
3. State Key Laboratory of Molecular Biology, Center for Excellence in Molecular Cell Science, Shanghai Institute of Biochemistry and Cell Biology, Chinese Academy of Sciences, Shanghai, China.
4. University of Chinese Academy of Sciences, Beijing, China.
5. School of Life Science, Hangzhou Institute for Advance Study, University of Chinese Academy of Sciences, Hangzhou, Zhejiang, China.

These authors contributed equally to this work: Shuolin Liu, Yaguang Bi, Tianting Han

*Correspondence: Ronggui Hu (coryhu@sibcb.ac.cn) or Yingmei Zhang (zhang.yingmei@zs-hospital.sh.cn) or Junbo Ge (ge.junbo@zs-hospital.sh.cn)

Supplementary Table S1. Patient Characteristics

Sample	Sex	Age (years)	Diagnosis
Control 1	M	18	Brain death
Control 2	F	24	Brain death
Control 3	M	33	Brain death
Control 4	M	29	Brain death
Control 5	F	31	Brain death
Control 6	M	35	Brain death
IHD 1	F	43	ICM
IHD 2	M	51	ICM
IHD 3	M	56	ICM
IHD 4	M	45	ICM
IHD 5	M	64	ICM
IHD 6	M	57	ICM

IHD, ischemic heart disease; ICM, ischemic cardiomyocytes.

SUPPLEMANTARY FIGURES AND FIGURE LEGENDS

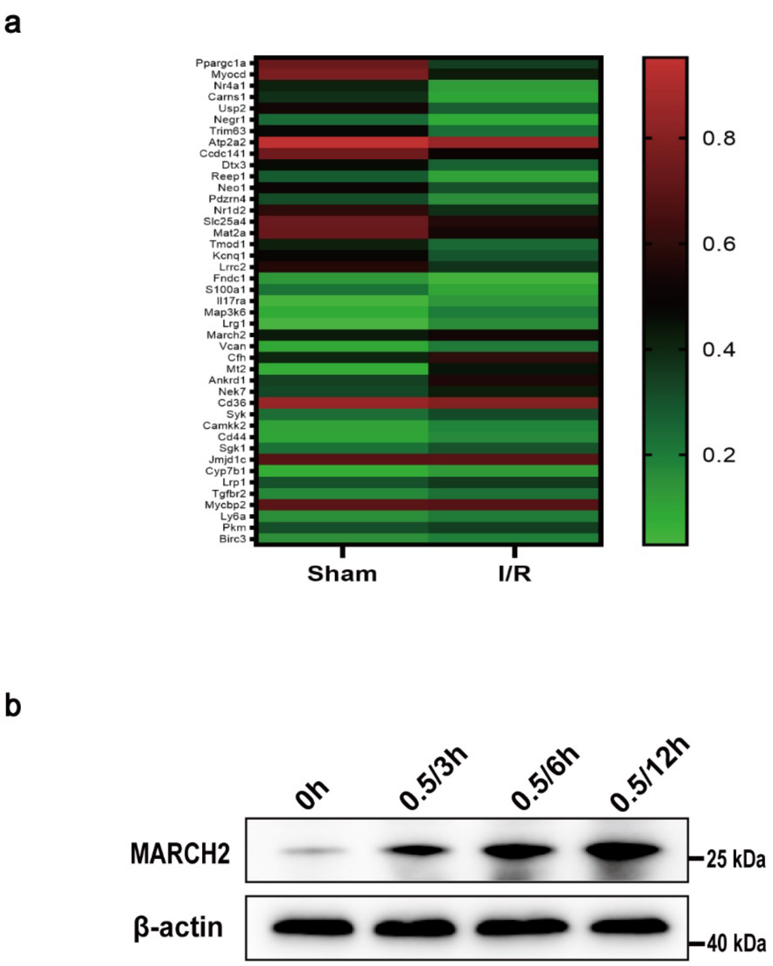

Supplementary Fig. S1. Differentially expressed genes and MARCH2 level after myocardial I/R injury. a Heatmaps showing the significantly altered genes after myocardial I/R injury. **b** Western blotting analysis of MARCH2 expression in primary adult mouse cardiomyocytes subjected to hypoxia and reoxygenation for different times.

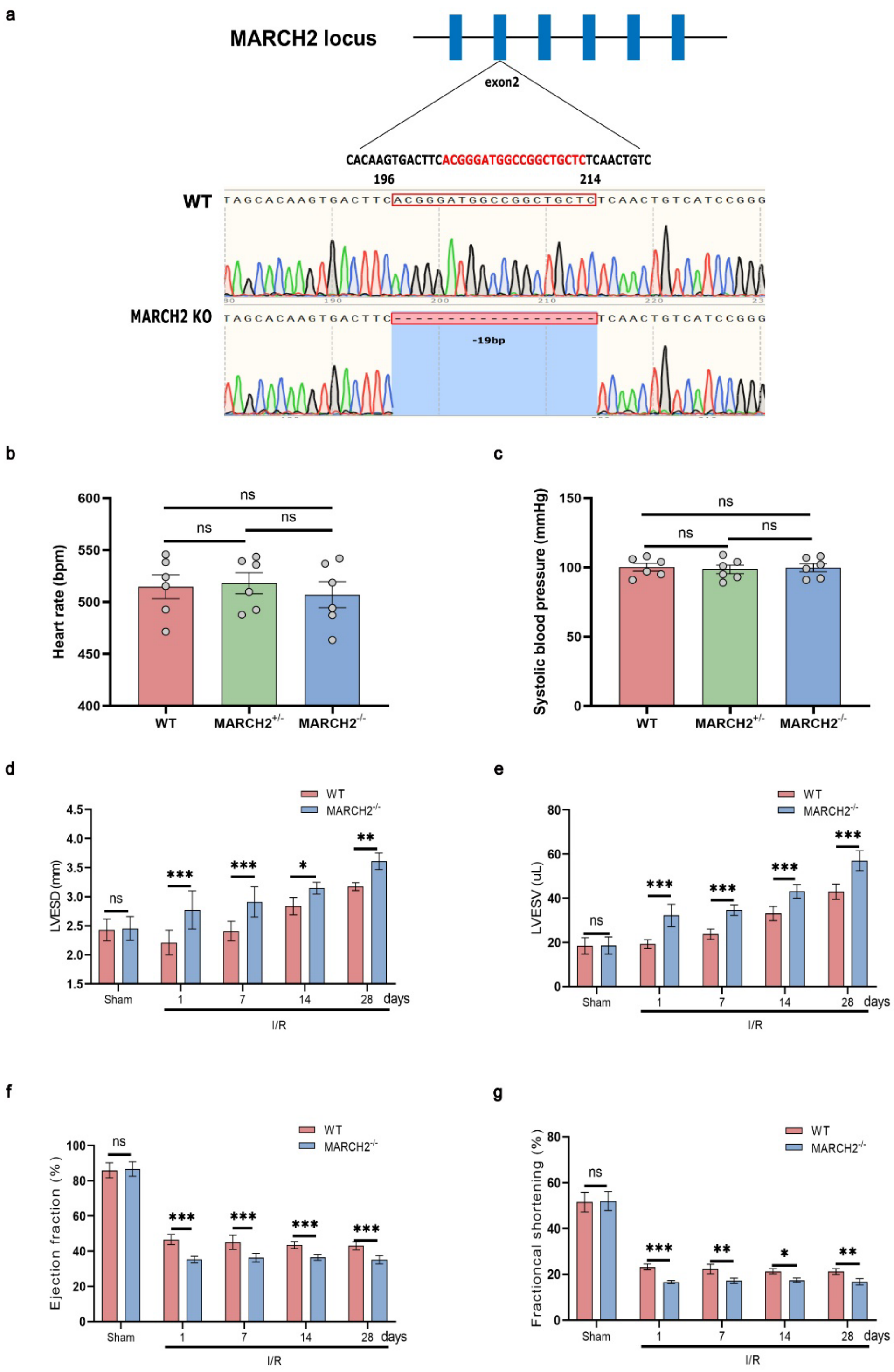

Supplementary Fig. S2. The generation of MARCH2 deletion mice (MARCH2 KO) and long-term cardiac function after myocardial I/R injury. a Generation of MARCH2 deletion mice. **b, c** Baseline heart rate (b) and blood pressure (c) of mice was measured by echocardiography and tail-cuff method. **d, e, f, g** Longitudinal assessment

of cardiac structure and function by echocardiography was performed after I/R injury. Left ventricular end systolic diameter (LVESD, d), left ventricular end systolic volume (LVESV, e), ejection fraction (EF, f), and fractional shortening (FS, g). n = 6 mice per group.

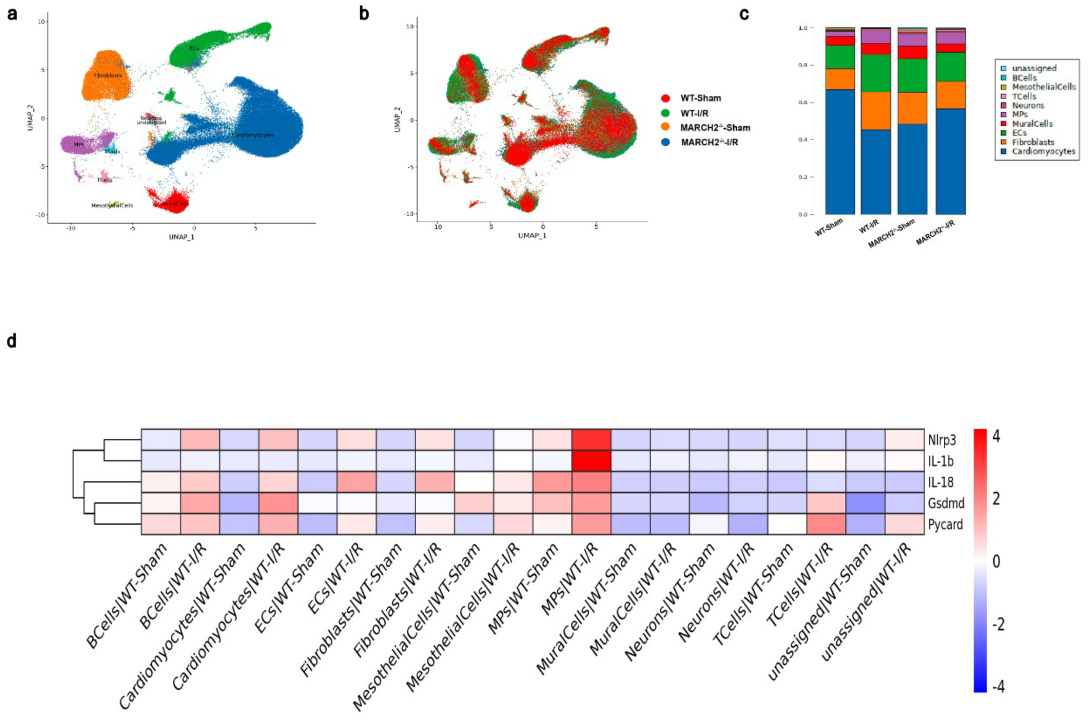

Supplementary Fig. S3 Cell types distribution and percentages of single cells. a Uniform Manifold Approximation and Projection (UMAP) showing 178274 single cells isolated from WT and MARCH2$^{-/-}$ mice after sham or I/R surgery. EC, endothelial cell. MP, macrophage. Cell types were identified based on the expression of known markers. **b, c** Cell types distribution (b) and percentages (c) for different groups were determined by the cluster of cells. **d** Single-cell RNA-seq of NLRP3 inflammasome assembly-related genes between WT-Sham group and WT-IR group in each cell type [cardiomyocytes, fibroblasts, endothelial cells (ECs), Mural cells, macrophages (MPs), neurons, T cells, mesothelial cells, and B cells].

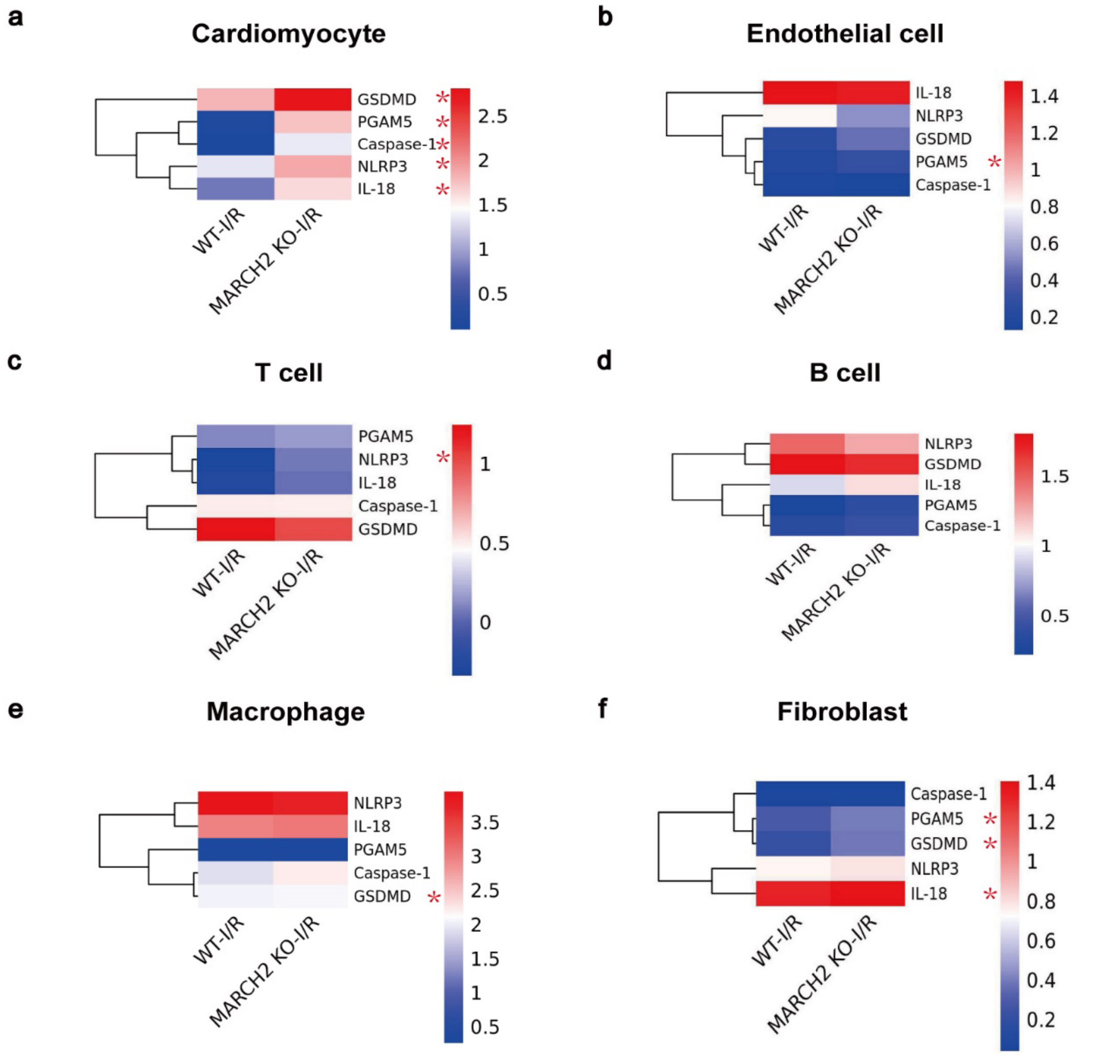

Supplementary Fig. S4. Single-cell RNA-seq of NLRP3 inflammasome assembly-related genes in different cell types. a, b, c, d Differentially expressed genes including PGAM5 (a), NLRP3 (b), GSDMD (c), Caspase-1 (d) between MARCH2 KO-IR group and WT-IR group in each cell type [cardiomyocytes, fibroblasts, endothelial cells (ECs), Mural cells, macrophages (MPs), neurons, T cells, mesothelial cells, and B cells]. Statistical differences were determined using Mann–Whitney U test. * represents $P<0.05$. MARCH2, Membrane-associated RING finger protein 2; PGAM5, phosphoglycerate mutase 5; NLRP3, NACHT, LRR and PYD domains-containing protein 3; GSDMD, Gasdermin-D.

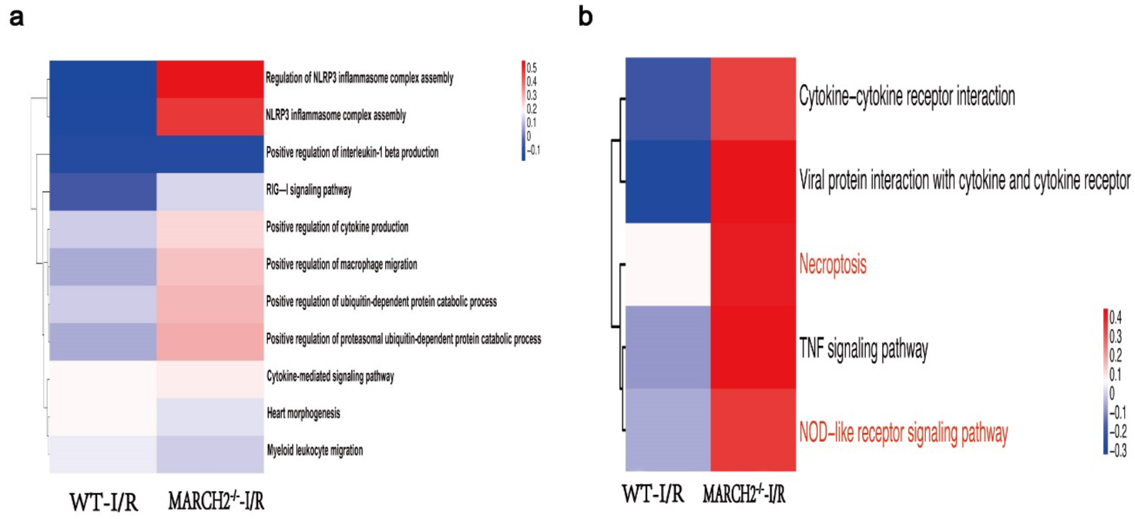

Supplementary Fig. S5. MARCH2 knockout up-regulated NLRP3 inflammasome assembly and pyroptosis in cardiomyocytes. The NLRP3 inflammasome assembly-related gene sets were quantified in cardiomyocytes. **a** Comparative gene set variation analysis (GSVA) of KEGG pathway between WT-I/R and MARCH2$^{-/-}$-I/R groups. **b** comparative gene set variation analysis (GSVA) of GO between WT-I/R and MARCH2$^{-/-}$-I/R groups. MARCH2, Membrane-associated RING finger protein 2; PGAM5, phosphoglycerate mutase 5; NLRP3, NACHT, LRR and PYD domains-containing protein 3; GSDMD, Gasdermin-D.

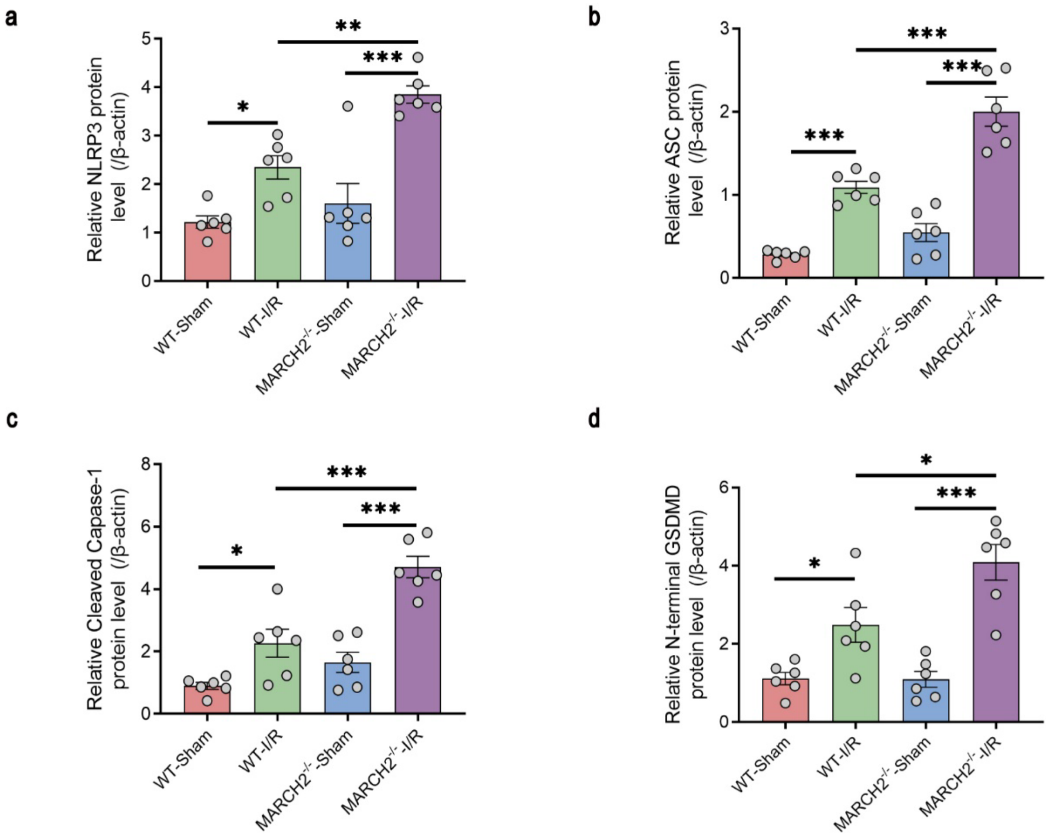

Supplementary Fig. S6. The expression levels of NLRP3 inflammasome pathway target genes in WT and MARCH2 KO mice subjected to I/R. a, b, c, d Quantitated analysis of NLRP3, ASC, Cleaved Caspase-1, and N-terminal GSDMD protein levels in WT and MARCH2 KO mice subjected to I/R. Data are shown as the means ± SEM. * $P < 0.05$, ** $P < 0.01$, *** $P < 0.001$. MARCH2, Membrane-associated RING finger protein 2; NLRP3, NACHT, LRR and PYD domains-containing protein 3; ASC, Apoptosis-associated speck-like protein containing a CARD; GSDMD, Gasdermin-D.

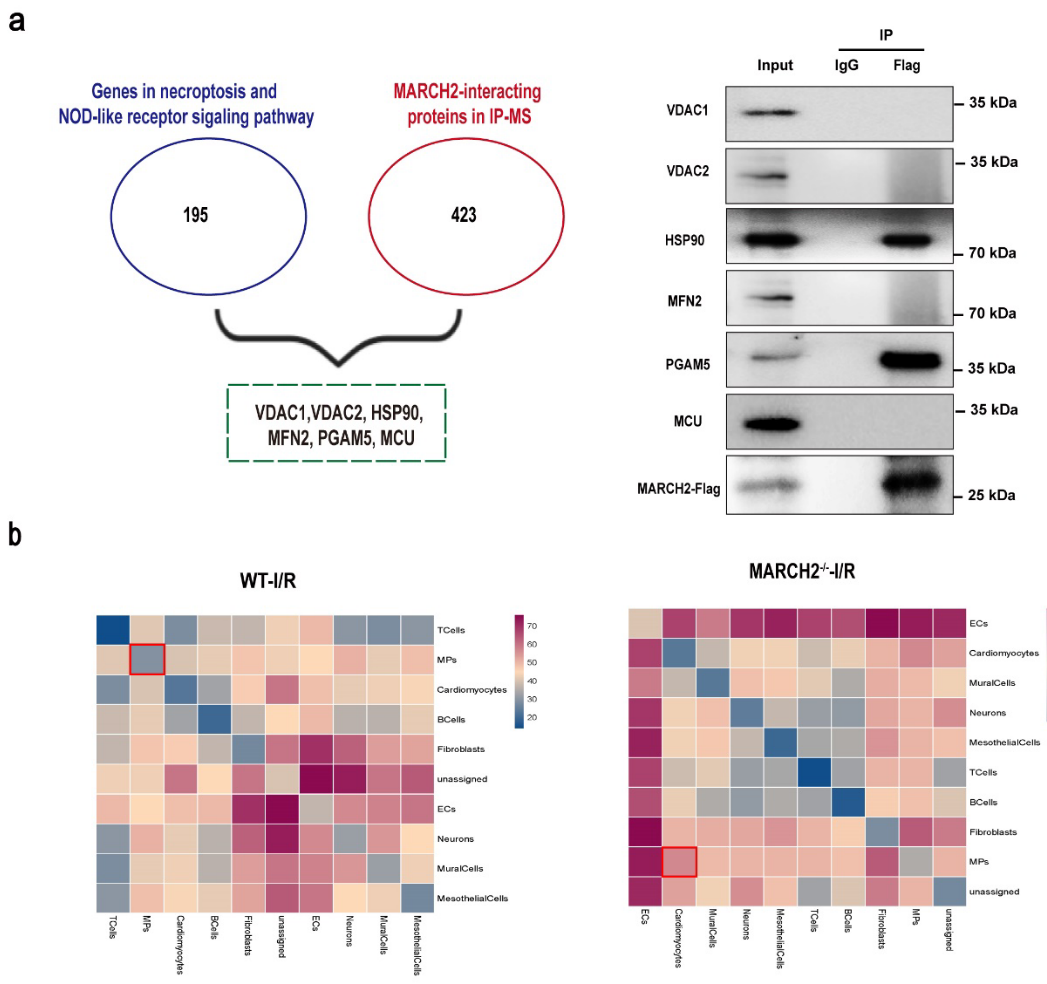

Supplementary Fig. S7. Validation of potential MARCH2-binding protein and cell-cell interactions in mouse heart following myocardial ischemia/reperfusion injury. a Schematic diagram showing the conjoint screening of genes both in necroptosis or NOD-like receptor signaling pathway (KEGG pathways HSA04217, HSA04621) and in mass spectrometry (MS). Validating potential MARCH2-binding protein in MS with immunoprecipitation in HL-1 cardiomyocytes. **b** Cell-cell interactions in WT-I/R and MARCH2$^{-/-}$-I/R groups were predicted based on ligand-receptor pairs by Cellphone DB. The interactions between macrophages (MPs) and cardiomyocytes were significantly increased in MARCH2$^{-/-}$-I/R group compared with WT-I/R group (red box).

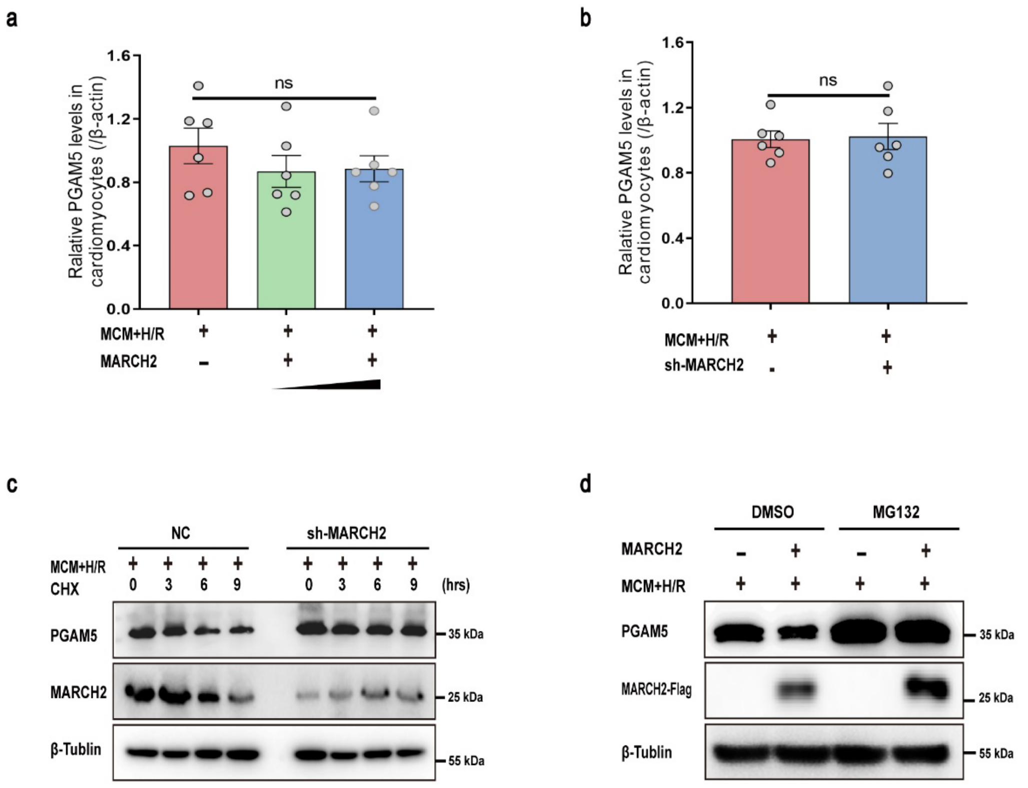

Supplementary Fig. S8. MARCH2 mediated the degradation of PGAM5 protein in NMCMs. a, b The mRNA levels of PGAM5 did not change when either overexpression (a) or knockdown (b) of MARCH2 in NMCMs treated with MCM+H/R (n = 6 for each group). The PGAM5 mRNA levels were analyzed by RT-PCR. **c** Representative Western blotting analysis of MARCH2 and PGAM5 protein levels. NMCMs were transfected with NC or sh-MARCH2 and then treated with cycloheximide (CHX; 30 μM) for indicated time periods in the setting of MCM+H/R treatment. **d** NMCMs were transfected with Vector or MARCH2 and were treated with or without MG132 (10 μM) in the setting of MCM+H/R treatment. Data are shown as the means ± SEM. MARCH2, Membrane-associated RING finger protein 2; PGAM5, phosphoglycerate mutase 5; NMCMs, neonatal mouse cardiomyocytes.

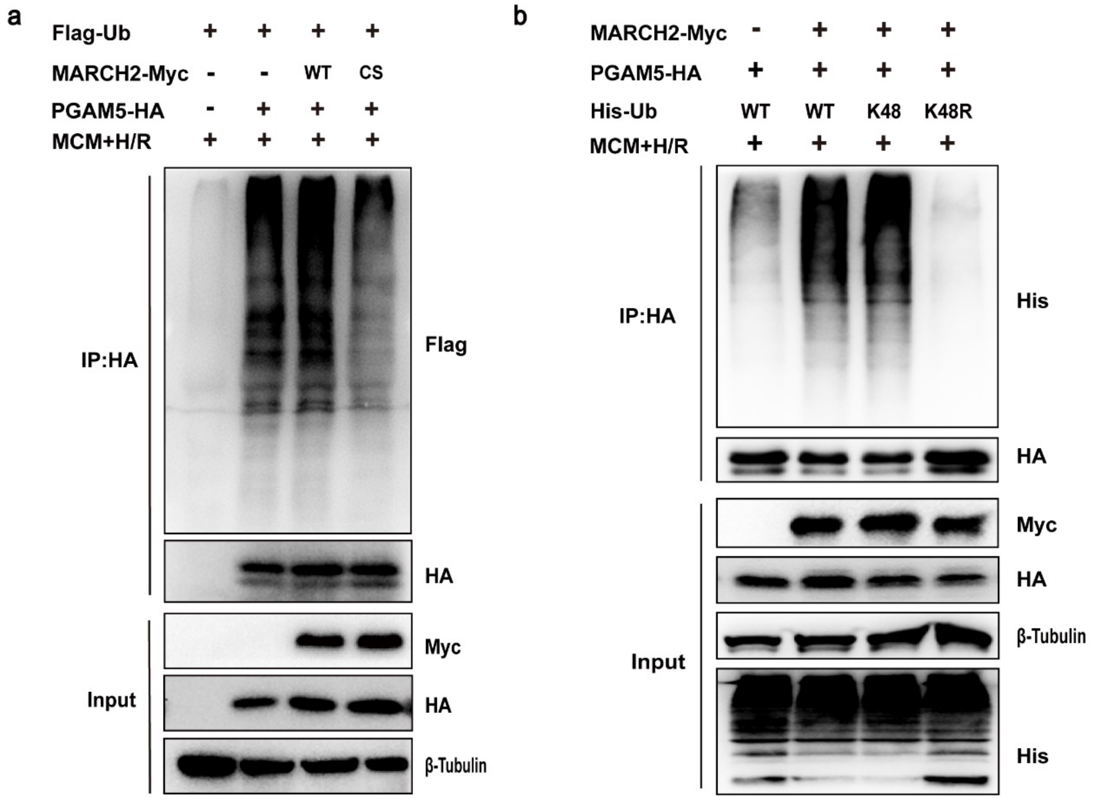

Supplementary Fig. S9. MARCH2 E3 ligase promotes the degradation of PGAM5 via K48-linked polyubiquitination. a MARCH2 but not its E3 ligase-inactive mutants mediates polyubiquitination of PGAM5. HL-1 cardiomyocytes were transfected with PGAM5-HA, Flag-Ub and MARCH2-myc, or MARCH2-CS-myc and treated with H/R. **b** Effects of the K48-only ubiquitin KR (Lys to Arg) mutant on MARCH2-mediated PGAM5 ubiquitination. HL-1 cardiomyocytes were transfected with the K48 ubiquitin and K48R mutants under H/R treatment. Immunoprecipitation analysis with anti-HA antibody and immunoblotting with antibody of anti-His and anti-HA.

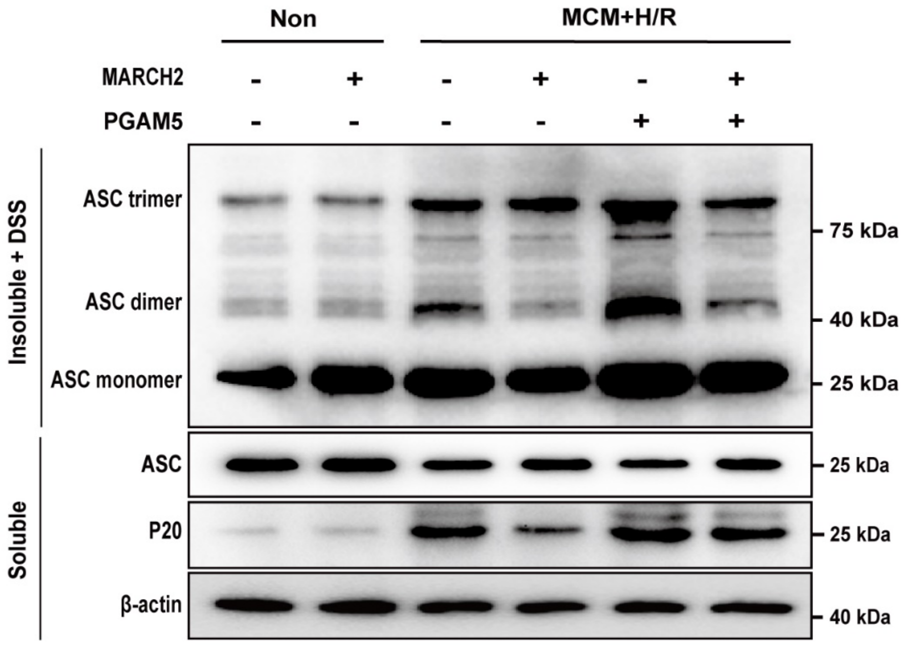

Supplementary Fig. S10. PGAM5 mediates the regulation of MARCH2 on ASC oligomerization. Neonatal mouse cardiomyocytes were subjected to cross-linking of detergent-insoluble fraction with DSS and the ASC oligomers were detected with ASC antibody.

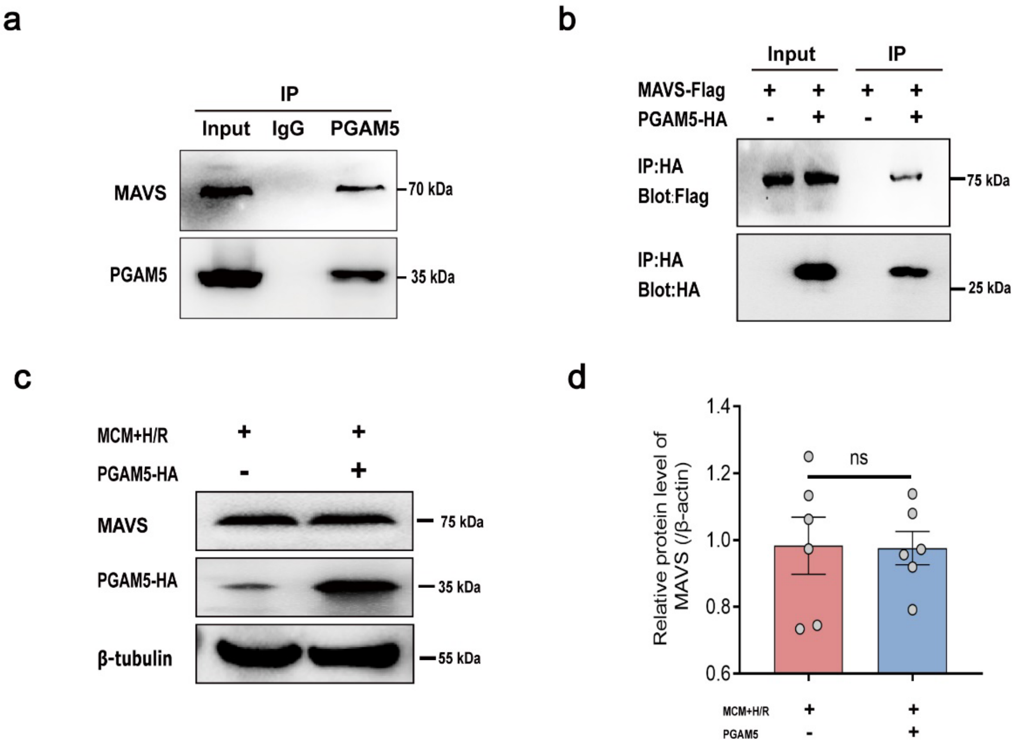

Supplementary Fig. S11. PGAM5 interacted with MAVS. a Endogenous immunoprecipitation of MAVS and PGAM5 in HL-1 cardiomyocytes. **b** Immunoprecipitation of HA-PGAM5 and Flag-MAVS in HL-1 cardiomyocytes. **c, d** Representative Western blotting image (c) and quantitated analysis (d) of MAVS expression in cardiomyocytes infected with vector or PGAM5 under MCM+H/R treatment. PGAM5, phosphoglycerate mutase 5; MAVS, Mitochondrial antiviral-signaling protein.

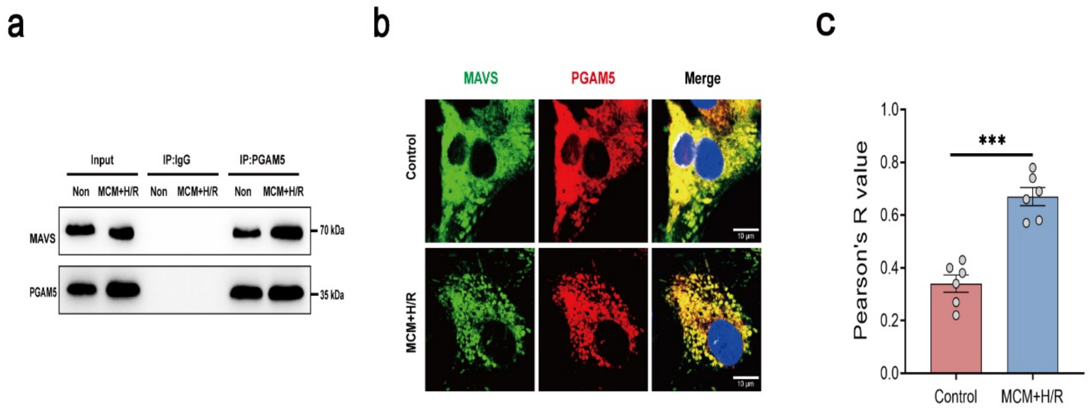

Supplementary Fig. S12. MCM+H/R induced a more robust interaction between endogenous PGAM5 and MAVS in cardiomyocytes. a Interaction between PGAM5 and MAVS was examined by IP-Western blot in neonatal mouse cardiomyocytes (NMCMs) with or without MCM+H/R treatment. **b** Representative immunofluorescence images of MAVS and PGAM5 in NMCMs. **c** Colocalization analysis of PGAM5-MAVS. Pearson's R value (no threshold) was calculated by ImageJ Fiji software; n=6 images from 3 biological replicates. Data are shown as the means ± SEM. *** P < 0.001. PGAM5, phosphoglycerate mutase 5; MAVS, Mitochondrial antiviral-signaling protein; H/R, hypoxia and reoxygenation; MCM, macrophage-conditioned medium.

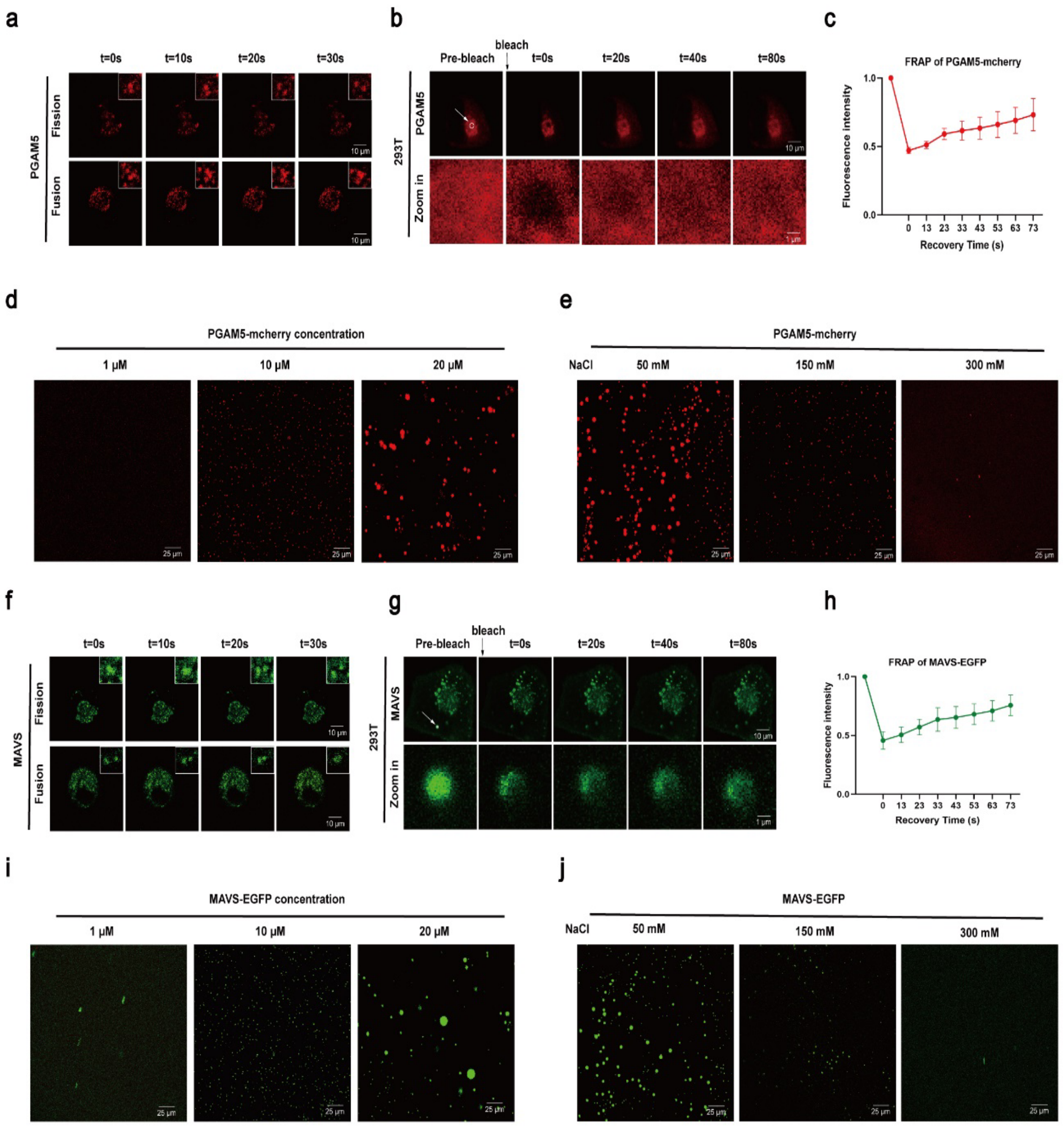

Supplementary Fig. S13. PGAM5-MAVS co-condensates forms in 293T cells under MCM+H/R treatment. a Time-lapse image of 293T cells expressing PGAM5-mcherry. PGAM5 condensate fission and fusion is presented in the boxes. **b** Fluorescence recovery after photobleaching (FRAP) analysis of PGAM5-mcherry condensate in 293T cells. **c** Quantification of FRAP in the bleached region of PGAM5-mcherry condensate, show as the mean±SD (n=6). **d** Cell-free phase separation assay of PGAM5-mcherry droplet formation at different concentrations. **e** Images of PGAM5-mcherry (25 μmol/L) droplet formation at different salt concentrations. **f** Time-lapse images of 293T cells expressing MAVS-EGFP. MAVS condensate fission

and fusion is presented in the boxes. **g** FRAP analysis of MAVS-EGFP condensate in 293T cells. **h** Quantification of FRAP in the bleached region of MAVS-EGFP condensate, show as the mean±SD (n=6). **i** Cell-free phase separation assay of MAVS-EGFP droplet formation at different concentrations. **j** Images of MAVS-EGFP (20 μmol/L) droplet formation at different salt concentrations.

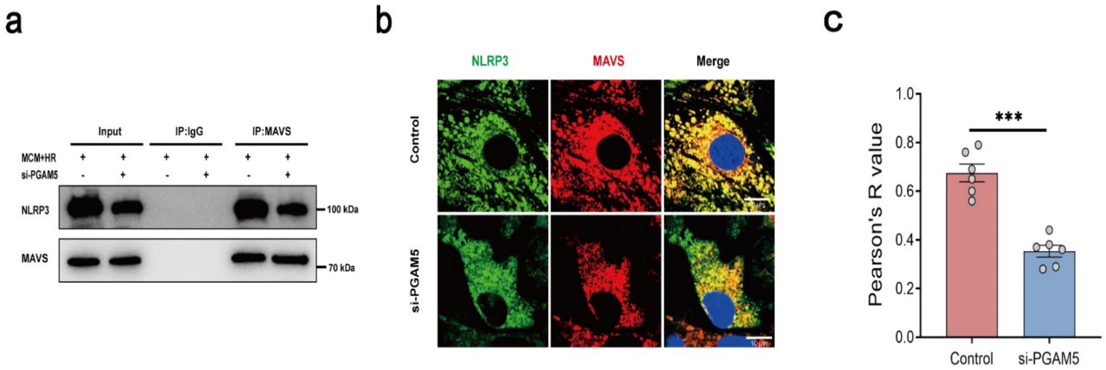

Supplementary Fig. S14. PGAM5 mediates the interaction between NLRP3 and MAVS in cardiomyocytes. a Interaction between NLRP3 and MAVS was examined by immunoprecipitation (IP)-Western blot in neonatal mouse cardiomyocytes (NMCMs) in the presence or absence of si-PGAM5 under MCM+H/R treatment. **b** Immunofluorescence of NLRP3 and MAVS in NMCMs with or without PGAM5 knockdown under MCM+H/R treatment. **c** colocalization analysis of NLRP3-MAVS. Pearson's R value (no threshold) was calculated by ImageJ Fiji software; n=6 images from 3 biological replicates. Data are shown as the means ± SEM. *** P < 0.001. PGAM5, phosphoglycerate mutase 5; MAVS, Mitochondrial antiviral-signaling protein; NLRP3, NACHT, LRR and PYD domains-containing protein 3; H/R, hypoxia and reoxygenation; MCM, macrophage-conditioned medium.

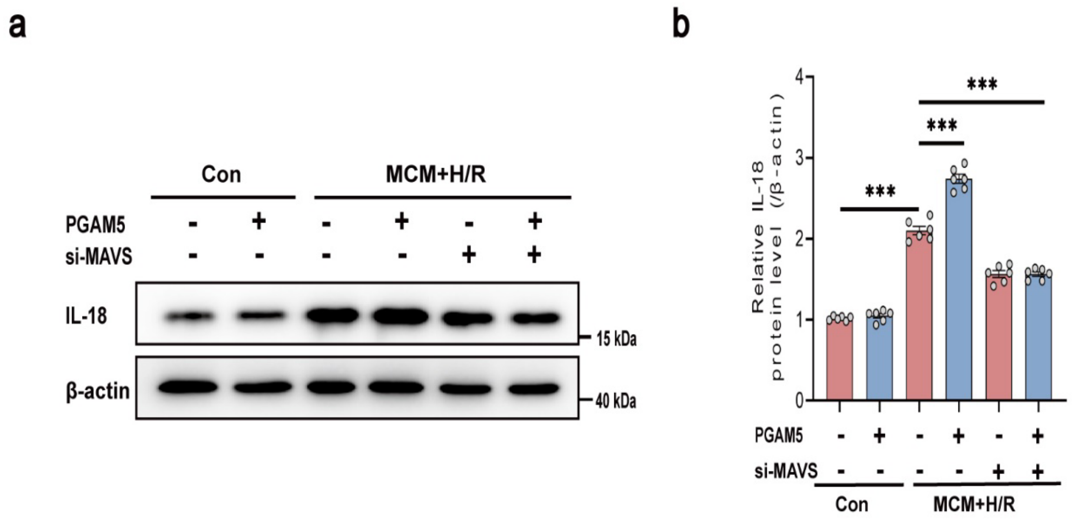

Supplementary Fig. S15. PGAM5 promotes MAVS-dependent IL-18 release in cardiomyocytes. a, b Representative Western blotting and quantitated analysis of IL-18 in cardiomyocytes infected with PGAM5 or si-MAVS under MCM+H/R treatment. PGAM5, phosphoglycerate mutase 5; MAVS, Mitochondrial antiviral-signaling protein; H/R, hypoxia and reoxygenation; MCM, macrophage-conditioned medium.

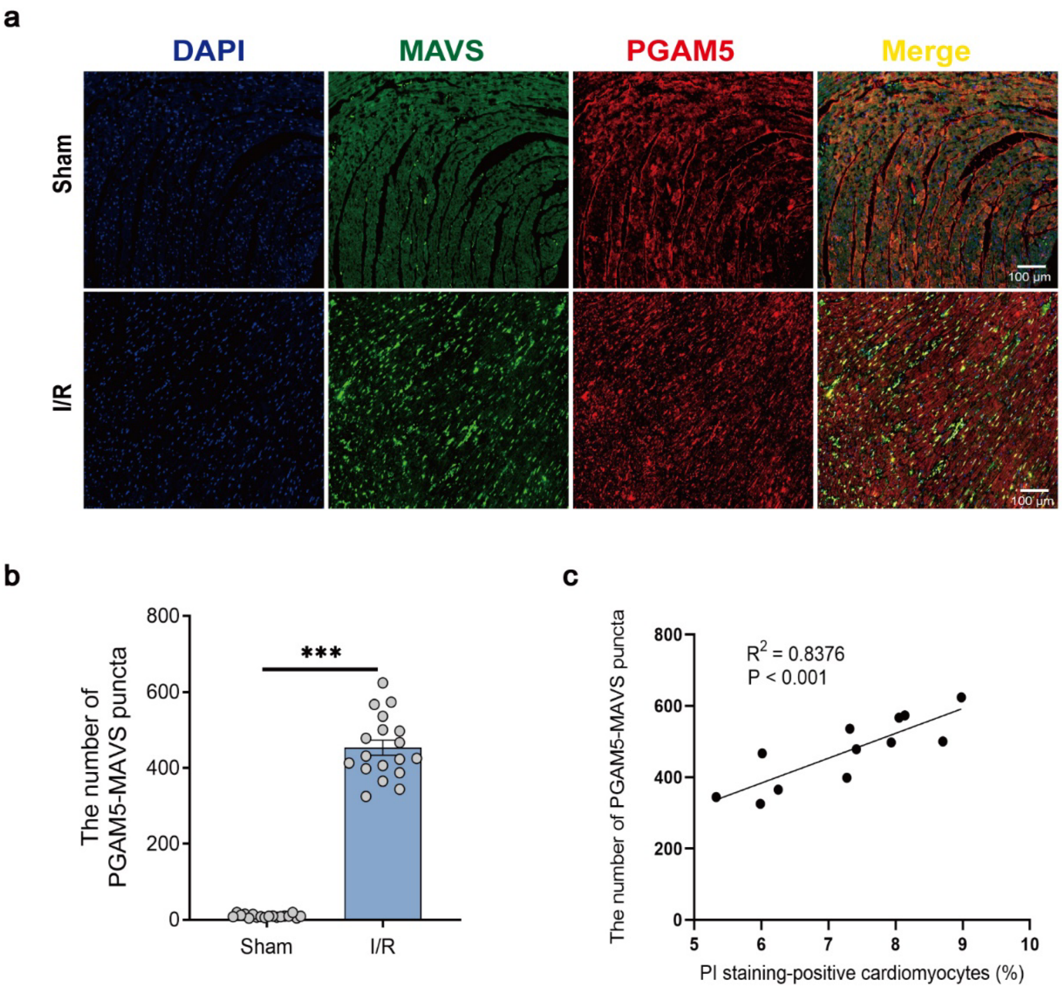

Supplementary Fig. S16. PGAM5-MAVS form puncta following myocardial I/R injury. a Immunofluorescence of PGAM5 (red) and MAVS (green) in the mouse hearts following sham or myocardial I/R injury. **b** The number of PGAM5-MAVS puncta in the mouse hearts following sham or myocardial I/R injury. N=18 sections from 6 mice. Data were analyzed by Mann-Whitney test. Data are shown as the means ± SEM. *** $P < 0.001$. **c** Pearson Correlation Coefficients between the number of PGAM5-MAVS puncta and PI staining cells in mouse hearts suffering myocardial I/R injury. PGAM5, phosphoglycerate mutase 5; MAVS, Mitochondrial antiviral-signaling protein.

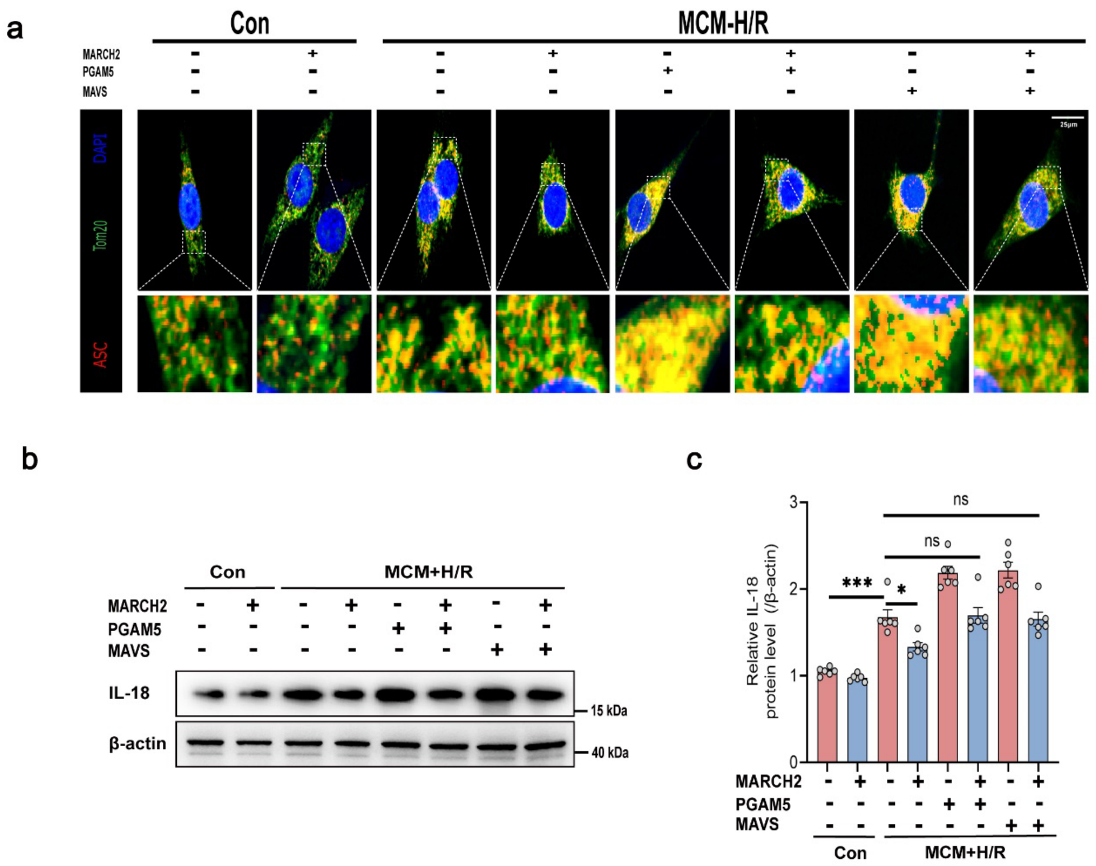

Supplementary Fig. S17. The regulation of MARCH2 on PGAM5-MAVS-NLRP3 inflammasome pathway in cardiomyocytes. **a** Immunofluorescence images of ASC and Tom20 colocalization in HL-1 cardiomyocytes with MARCH2, PGAM5 or MAVS overexpression following MCM+H/R challenge. **b, c** Representative Western blotting and quantitated analysis of IL-18 in cardiomyocytes infected with MARCH2, PGAM5, or MAVS under MCM+H/R treatment. MARCH2, Membrane-associated RING finger protein 2; PGAM5, phosphoglycerate mutase 5; MAVS, Mitochondrial antiviral-signaling protein; ASC, Apoptosis-associated speck-like protein containing a CARD.

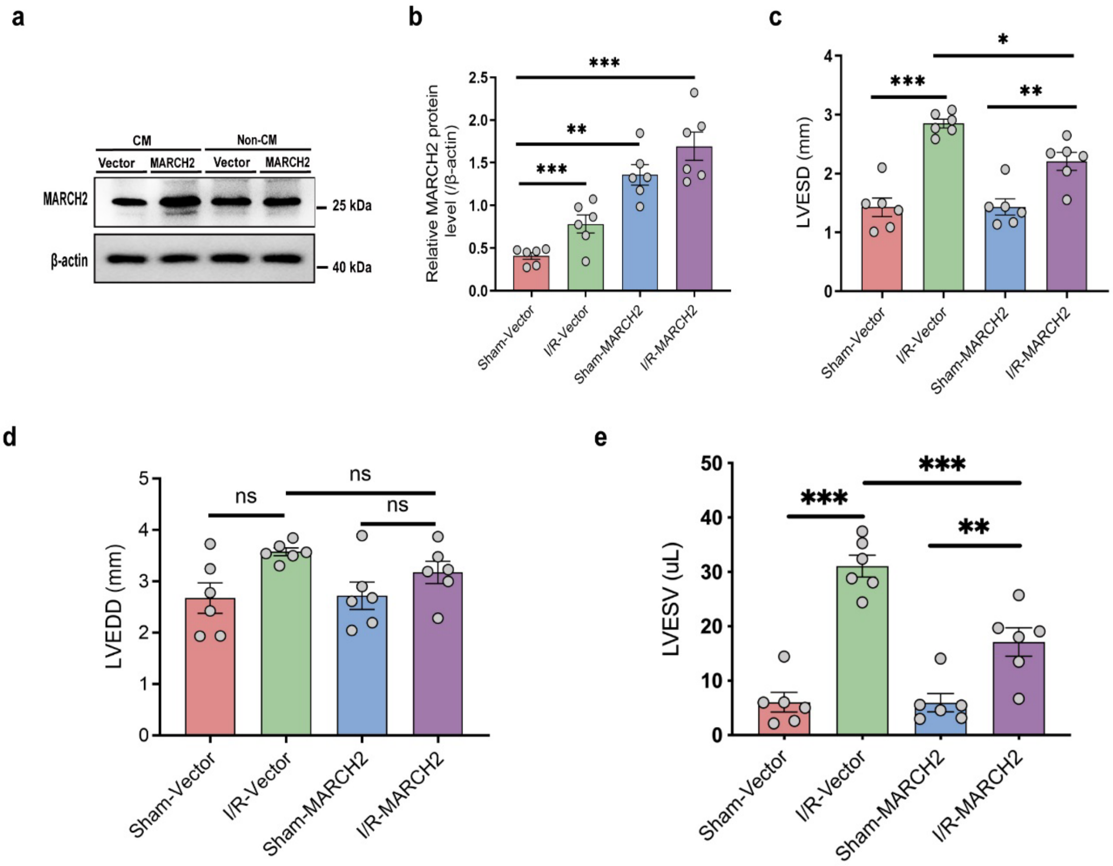

Supplementary Fig. S18. Overexpression MARCH2 protected heart from myocardial I/R injure and PGAM5 mediates its regulation on NLRP3 assembly in heart. a, b Represent western blot image (a) and quantitative analysis (b) demonstrated the AAV9 efficiency of MARCH2 expression in heart. **c, d, e** Quantitative analysis of echocardiographic measurements performed in AAV9-cTnT-NC or AAV9-cTnT-MARCH2 mice subjected to I/R (45min/24 hours) injury (n=6 mice per group). Left ventricular end systolic diameter (LVESD, c), left ventricular end diastolic diameter (LVEDD, d), left ventricular end systolic volume (LVESV, e). Data are shown as the means ± SEM. * $P < 0.05$, ** $P < 0.01$, *** $P < 0.001$. MARCH2, Membrane-associated RING finger protein 2.

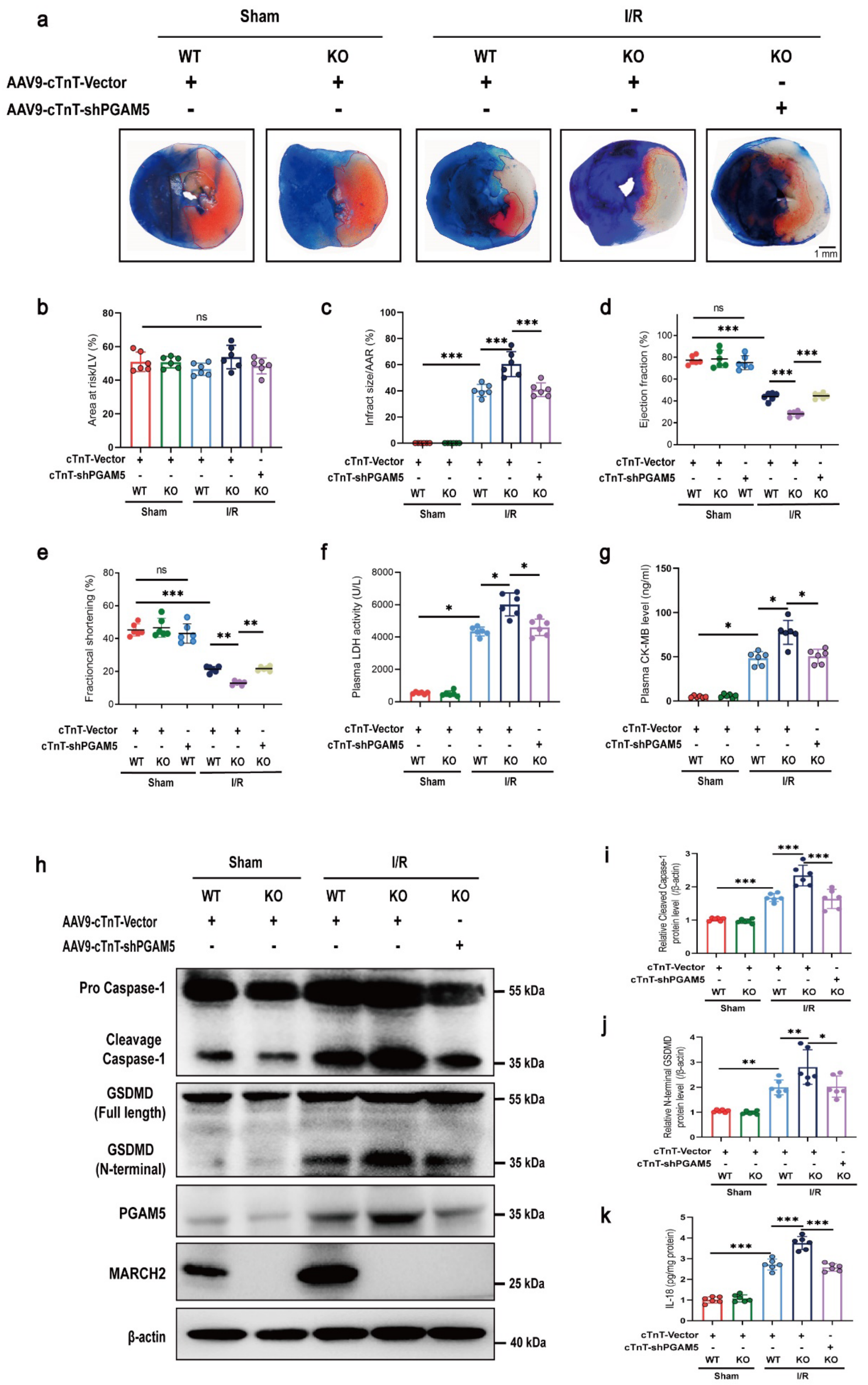

Supplementary Fig. S19. Cardiomyocyte-specific PGAM5 knockdown in

MARCH2 KO mice lead to alleviation of the myocardial infarction area, myocardial function, and inflammatory response. a Before subjected to I/R, WT and MARCH2 KO mice were injected with AAV9-cTnT-shPGAM5 or AAV9-cTnT-Vector (2×10^{11} V.g/mouse) for 3 weeks by tail vein injection. TTC/Evans Blue staining is used to depict infracted area. **b** Ratios of area at risk (AAR) to left ventricular (LV) area; **c** Infarct area normalized to AAR; **d, e** Echocardiographic assessment of ejection fraction (EF, d) and FS (fractional shortening, e) in the indicated groups. **f, g** LDH release (f) and CK-MB activity (g) in mouse serum with and without myocardial I/R. **h, i, j** Representative Western blotting image (h) and quantitated analysis of caspase-1 (Procasp1; cleaved caspase-1, i) and GSDMD (Full length; N-terminal, j) in hearts of indicated group mice subjected to I/R (45 minutes/6 hours). **k** The IL-18 release was measured by ELISA in cardiac tissues of the indicated group mice subjected to I/R (45 minutes/9 hours). Data are shown as the means ± SEM. *P < 0.05. MARCH2, Membrane-associated RING finger protein 2; PGAM5, phosphoglycerate mutase 5.

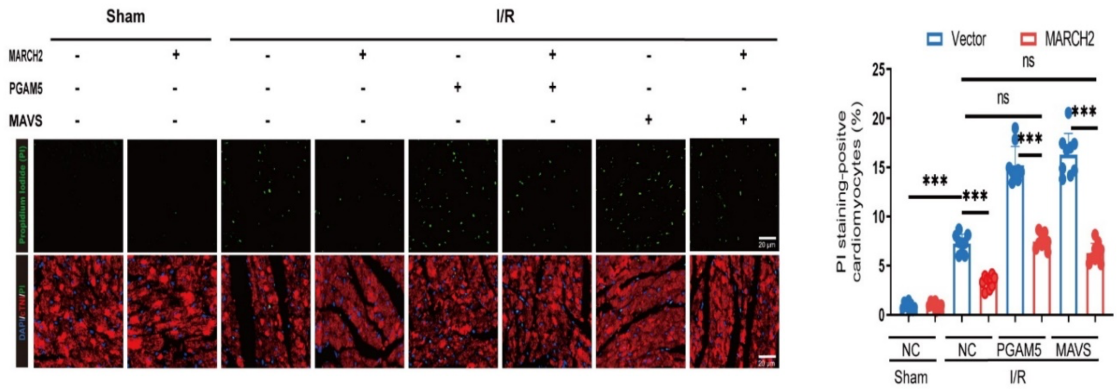

Supplementary Fig. S20. Propidium iodide (PI) stained images of myocardial sections from mice subjected to I/R. Green: PI-positive nuclei; Red: cTnI-stained cardiomyocytes; blue, DAPI-stained nuclei; Scale bar = 20 μm. *** P < 0.001. MARCH2, Membrane-associated RING finger protein 2; PGAM5, phosphoglycerate mutase 5; MAVS, Mitochondrial antiviral-signaling protein; I/R, ischemic and reperfusion.
